# COCOA: coordinate covariation analysis of epigenetic heterogeneity

**DOI:** 10.1186/s13059-020-02139-4

**Published:** 2020-09-07

**Authors:** John T. Lawson, Jason P. Smith, Stefan Bekiranov, Francine E. Garrett-Bakelman, Nathan C. Sheffield

**Affiliations:** 1 Department of Biomedical Engineering, University of Virginia, Charlottesville, VA USA; 2 Center for Public Health Genomics, University of Virginia, Charlottesville, VA USA; 3 Department of Biochemistry and Molecular Genetics, University of Virginia, Charlottesville, VA USA; 4 Department of Medicine, University of Virginia, Charlottesville, VA USA; 5 University of Virginia Cancer Center, Charlottesville, USA

**Keywords:** Epigenetics, DNA methylation, Chromatin accessibility, Principal component analysis, Dimensionality reduction, Data integration, Cancer, EZH2, Multi-omics

## Abstract

A key challenge in epigenetics is to determine the biological significance of epigenetic variation among individuals. We present Coordinate Covariation Analysis (COCOA), a computational framework that uses covariation of epigenetic signals across individuals and a database of region sets to annotate epigenetic heterogeneity. COCOA is the first such tool for DNA methylation data and can also analyze any epigenetic signal with genomic coordinates. We demonstrate COCOA’s utility by analyzing DNA methylation, ATAC-seq, and multi-omic data in supervised and unsupervised analyses, showing that COCOA provides new understanding of inter-sample epigenetic variation. COCOA is available on Bioconductor (http://bioconductor.org/packages/COCOA).

## Introduction

Epigenetic data is inherently high-dimensional and often difficult to interpret. Because of the high dimensionality, it is common to group individual genomic loci into collections that share a functional annotation, such as binding of a particular transcription factor [1–3]. These genomic locus collections, or region sets, are analogous to the more common gene sets, but relax the constraint that data must be gene-centric. While gene set approaches may be applied to epigenetic data by linking regions to nearby genes [4], this linking process is ambiguous and loses information because a regulatory locus may affect the expression of multiple genes or more distant genes. Alternatively, a region-centric approach is often more appropriate for epigenetic data, and there are now many region-based databases and analytical approaches [1, 2, 5–7], such as using region set databases for enrichment analysis [1, 7, 8] or to aggregate epigenetic signals from individual samples across regions to assign scores of regulatory activity to individual samples or single cells [2, 3, 6, 9].

Region-based methods have provided complementary ways to annotate and understand epigenomic data, but they suffer from three drawbacks: First, it is common to ignore covariation between the epigenetic signal and continuous patient phenotypes, relying instead on differential signals between discrete sample groups. This approach loses information about the differences among samples within a group. Second, the use of discrete cutoffs for identifying significant epigenetic differences between samples loses information about the strength of covariation between epigenetic features and sample phenotype. Third, existing approaches are generally specific to certain scenarios (e.g., unsupervised analysis) or data types (e.g., ATAC-seq) and therefore do not provide a generally applicable framework for covariation-based analysis.

Here, we present Coordinate Covariation Analysis (COCOA), a method for annotating epigenetic variation across individuals using region sets. COCOA offers several advantages compared to existing methods: First, COCOA provides a flexible framework that supports both supervised and unsupervised analysis. Second, for supervised analysis, COCOA leverages covariation information by allowing continuous sample phenotypes as well as discrete groups. Third, COCOA incorporates epigenetic signal values instead of using binarized values (i.e., significant or not significant), further taking advantage of the covariation information. Finally, COCOA works with any epigenetic data that have a numerical value associated with genomic coordinates, such as DNA methylation data, chromatin accessibility data, or even multi-omics data. Importantly, no such tool that leverages covariation of epigenetic signal across samples to annotate epigenetic variation previously existed for DNA methylation data. To demonstrate COCOA’s utility, we applied it in three unsupervised analyses with DNA methylation, ATAC-seq, and multi-omics data, and a supervised analysis of DNA methylation and cancer stage. We found that across multiple data types and biological systems, COCOA is able to identify promising biological sources of epigenetic heterogeneity across sample populations.

## Results and discussion

### An overview of COCOA

**Fig. 1:**
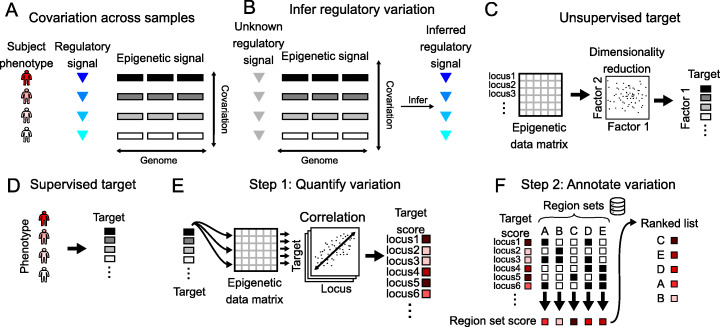
Overview of COCOA. **a** A regulatory signal may covary with the epigenetic signal in the genomic regions it regulates. **b** Covariation of the epigenetic signal in coregulated regions across individuals can be used to infer variation in the regulatory signal. **c** COCOA can be used with an unsupervised target variable (latent factor), or **d** with a supervised target variable (phenotype). **e** The first step is to quantify the relationship between the target variable and the epigenetic data at each locus, resulting in a score for each locus. **f** The second step is to annotate variation using a database of region sets. Each region set is scored to identify the region sets most associated with covariation between the epigenetic signal and the target variable. These top region sets can yield insight into the biological significance of the epigenetic variation

COCOA is an approach to understanding epigenetic variation among samples. COCOA derives its annotation power from a database of region sets that are grouped by function. This choice is rooted in the observation that a single effector, such as a transcription factor, often regulates many regions across the genome. Because the regions are coregulated, their epigenetic signal may covary across samples according to the activity of the effector (Fig. 1a), which can then be used to infer the activity of the effector (Fig. 1b). This principle of covariation of coregulated loci or genes has been leveraged by other methods related to gene regulation [2, 3, 9–13]. To distinguish small differences among samples in the activity level of the effector, COCOA boosts statistical power by aggregating signal in region sets [3].

COCOA uses this aggregated region set approach to annotate the underlying source of epigenetic variation that relates to a “target variable,” which can be either an unsupervised variable, like the primary latent factors in the data (Fig. 1c), or a supervised variable, like the phenotype of interest (Fig. 1d). COCOA annotates the inter-sample variation in the target variable by identifying region sets with variation patterns in epigenetic data that match the variation in the target variable. After a target variable is chosen, COCOA analysis consists of two main steps: first, for each locus, it computes the association of the inter-sample epigenetic variation with the target variable (Fig. 1e) and, second, it uses those associations to score a database of region sets (Fig. 1f). COCOA uses a permutation test to evaluate the statistical significance of each region set score. The result is a list of region sets ranked by how well the epigenetic signals in the region set correlate with the target variable. Highly scoring region sets have epigenetic signal that covaries across patients in the same way as the target variable, tying the functional annotation of the region set to the observed phenotypic variation.

### COCOA annotates inter-sample variation in breast cancer DNA methylation data

We first evaluated COCOA in an unsupervised analysis to determine if COCOA could identify and annotate a driving source of variation. We applied COCOA to DNA methylation data from breast cancer patients in The Cancer Genome Atlas (TCGA). In breast cancer, estrogen receptor (ER) status is a major prognostic factor and is known to be associated with a specific DNA methylation profile [14, 15]. We first used Principle Component Analysis to identify the top four Principal Components (PCs), which we used as the target variables, and asked whether COCOA would be able to identify ER as an important source of inter-sample variation using only the DNA methylation data, without requiring the samples’ ER status.

**Fig. 2:**
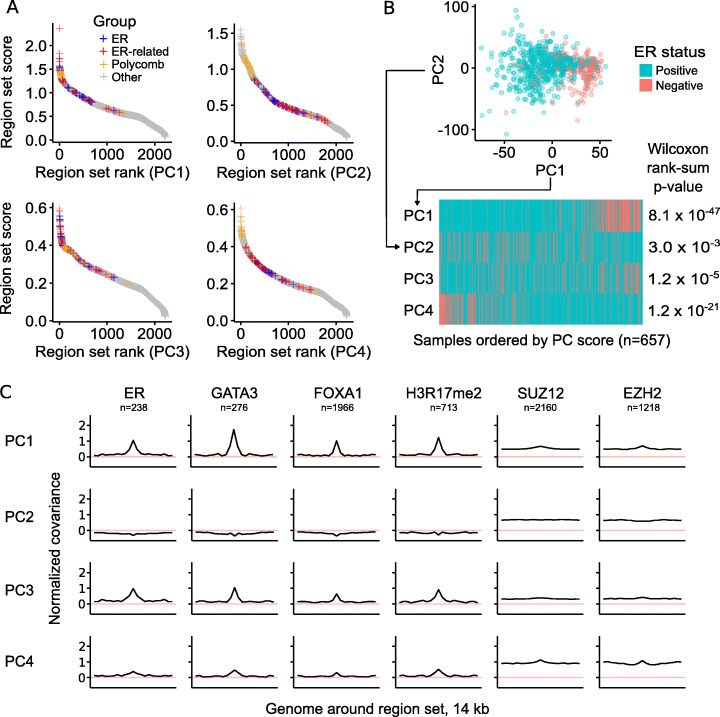
COCOA identifies sources of DNA methylation regulatory variation. **a** The COCOA score for each region set, ordered from highest to lowest. The ER-related group includes GATA3, FOXA1, and H3R17me2. The polycomb group includes EZH2 and SUZ12. **b** The association of PC scores with ER status for PCs 1–4 based on a Wilcoxon rank-sum test. **c** Meta-region profiles of several of the highest scoring region sets from PC1 (GATA3, ER, H3R17me2) and two polycomb group proteins (EZH2, SUZ12). Meta-region profiles show covariance between PC scores and the epigenetic signal in regions of the region set, centered on the regions of interest. A peak in the center indicates that DNA methylation in those regions covaries with the PC specifically around the sites of interest. The number of regions from each region set that were covered by the epigenetic data in the COCOA analysis (panel **a**) is indicated by “*n*”

COCOA identified a strong ER-associated signature for Principal Component 1 (PC1). This signature included many ER-binding region sets as top hits, indicating that variation of the DNA methylation in these ER-binding regions is associated with PC1 (Fig. 2a, Additional file 1: Table S1). We also identified variation in region sets for FOXA1 and GATA3, which are known to be associated with ER status [14, 15] (Additional file 1: Table S1). Furthermore, COCOA found the ER-associated histone modification H3R17me2 among the top scoring region sets [16] (Additional file 1: Table S1). When we test the association of each PC with ER status, PC1 scores have a highly significant association with ER status (*p* < 10^−46^, Wilcoxon rank-sum test), whereas PC2 and PC3 are less associated (Fig. 2b). Therefore, COCOA clearly identified ER-related variation as relevant for the primary axis of inter-sample variation, despite not having access to ER status information. We found that PC4 was also associated with ER status to a lesser extent (*p* < 10^−20^). For PC4, COCOA identified regions with repressive chromatin marks, including binding sites for polycomb components EZH2 and SUZ12 and repressive histone modifications H3K27me3 and H3K9me3 (Fig. 2a, Additional file 2: Fig. S1). Previous studies have linked polycomb expression to breast cancer: higher EZH2 expression is associated with ER− breast cancer [17, 18], EZH2 interacts with the repressor of estrogen activity (REA) protein [19], and Suz12-binding sites have DNA methylation differences between ER+ and ER− breast cancer [14]. Therefore, PC4 represents an additional aspect of ER-related epigenetic variation. PC2 and PC3 had weaker associations with ER status (*p* < 0.01 and *p* < 10^−4^ respectively); for PC3, the highest-ranking PC3 region sets include some ER-related region sets along with hematopoietic region sets (Additional file 1: Table S1). The hematopoietic region sets may represent inter-sample variation in the immune component of the tumors since breast cancer subtypes have been reported to be associated with differing immune cell profiles [20]. In summary, these results demonstrate that COCOA was able to identify relevant sources of inter-sample variation without requiring known sample groups and therefore reveal COCOA’s usefulness for unsupervised analysis of DNA methylation data.

To visualize the inter-sample variation that drives the top region sets identified by COCOA, COCOA can also plot DNA methylation in a region set, ordered by PC value (Additional file 2: Fig. S2). Using this approach, we visualized how the DNA methylation in ER-related regions varies along PC1, demonstrating clear covariation across regions that drives the region set rankings (Additional file 2: Fig. S2). To further confirm the specificity of the region sets, COCOA can also plot variation in broader genomic regions around the regions of interest. We found that the DNA methylation close to the transcription factor-binding regions shows stronger covariation with the PC score than DNA methylation in the surrounding genome (Fig. 2c). This visualization of specificity of the covariation to the binding regions provides additional evidence of the association between the PC and region set. Other high-ranking transcription factors also showed this specificity (Fig. 2c, Additional file 2: Fig. S3). Some histone modifications, such as H3K9me3 and H3K27me3, where DNA methylation levels had high covariation with the PC showed broader regions of elevated covariation (Additional file 2: Fig. S3). Overall, these visualization functions reveal aspects of epigenetic variation in the top region sets that could not be captured by a single region set score.

### COCOA annotates regulatory variation in ATAC-seq data

**Fig. 3:**
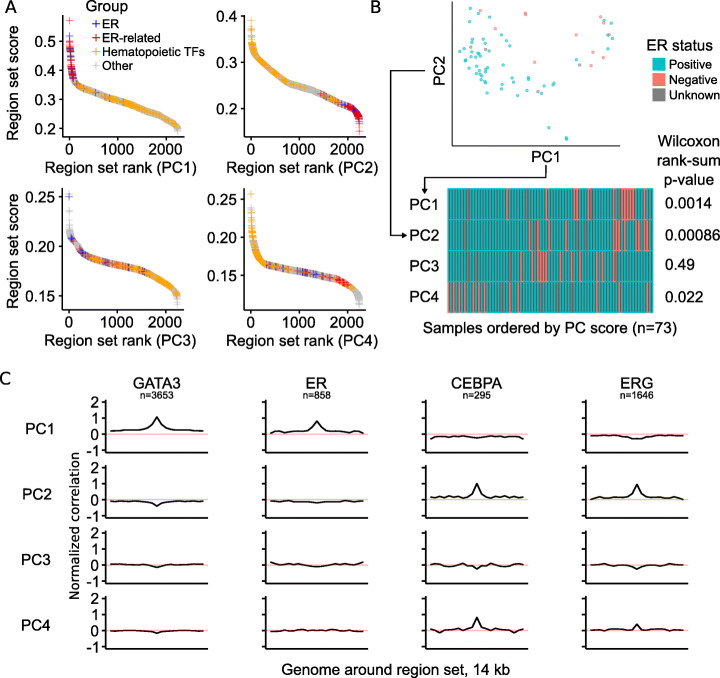
COCOA can be used for region-based data such as ATAC-seq. **a** The COCOA score for each region set, ordered from highest to lowest. The ER-related group includes GATA3, FOXA1, and H3R17me2. For definition of the hematopoietic TF group, see the section “Region set database” in the “Methods” section. **b** The association of PC scores with ER status for PCs 1–4 based on a Wilcoxon rank-sum test. **c** Meta-region profiles of the two highest scoring region sets from PC1 (GATA3, ER) and PC2 (CEBPA, ERG). Meta-region profiles show correlation between PC scores and the epigenetic signal in regions of the region set, centered on the regions of interest. A peak in the center indicates that chromatin accessibility in those regions correlates with the PC specifically around the sites of interest. The number of regions from each region set that were covered by the epigenetic data in the COCOA analysis (panel **a**) is indicated by “*n*”

Next, we asked whether COCOA could be applied to ATAC-seq data. Unlike DNA methylation data, which annotates individual nucleotides, ATAC-seq data is summarized by accessibility values at “peak” regions [21]. COCOA handles either data type. To demonstrate the region-type analysis, we ran COCOA with ATAC-seq data from TCGA breast cancer patients [21], expecting that ER-related region sets would be among our top results, similar to the DNA methylation data. As before, we used PCA on the ATAC-seq data and then applied COCOA to annotate the sources of variation for each PC. We identified many of the same region sets to be associated with epigenetic variation, despite far fewer samples (657 vs 73). We found ER-related region sets to be among the top ranked results for PC1 (Fig. 3a, Additional file 1: Table S2). PC2 was characterized by high-ranking hematopoietic transcription factors (Fig. 3a, Additional file 1: Table S2), once again potentially representing inter-sample variation in the immune component of the tumors [20], as in PC3 of the DNA methylation data. A few other top PCs including PC4 also had high-ranking hematopoietic transcription factors (Fig. 3a, Additional file 2: Fig. S4). Consistent with our results, visual inspection of the chromatin accessibility signal in top ER-related and hematopoietic region sets also revealed correlation between the signal and PC scores for the PCs in which the region sets were highly ranked (Additional file 2: Fig. S5). Polycomb region sets did not rank as prominently for the ATAC-seq data as for the DNA methylation data, but there were several polycomb region sets in the top 10% of region set scores for PC4 (Additional file 2: Fig. S6, Additional file 1: Table S2). These results are consistent with variation in ER status, which is significantly associated with PC1 and PC2 (*p* < 0.01, Wilcoxon rank-sum test, Fig. 3b) and to a lesser extent PC4 (*p* < 0.05). Visualization of the correlation between each PC and the ATAC-seq signal in the top region sets also shows specificity to the transcription factor-binding regions compared to the surrounding genome (Fig. 3c). Thus, COCOA can identify meaningful sources of variation in ATAC-seq data, providing a novel tool for regulatory analysis of ATAC-seq data.

### COCOA identifies regulatory variation in multi-omics integration

**Fig. 4:**
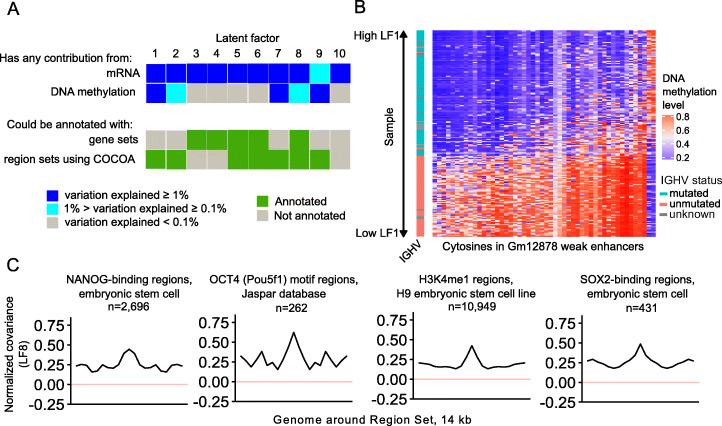
COCOA can be applied to multi-omics analyses that include epigenetic data. **a** COCOA can annotate latent factors that were not annotated by a gene set approach. In the top of panel **a**, dark blue indicates that the data type explained at least 1% of the variation of the latent factor while light blue indicates that the data type explained between 0.1 and 1% of the variation. Gray indicates less than 0.1% explained. In the bottom of panel **a**, green indicates that at least one statistically significant gene set or region set was found for the latent factor and gray indicates no significant gene or region sets were found. **b** COCOA identifies an enhancer region set from a transformed B lymphocyte cell line where DNA methylation is correlated with latent factor 1 and IGHV mutation status, a marker of mature B cells that have undergone somatic hypermutation. The 50 CpGs with the highest absolute correlation with LF1 from the region set are shown. **c** Meta-region profiles show covariation between DNA methylation and LF8 score in certain regions bound by transcription factors functional in stem cell biology and by H3K4me1 in a stem cell line compared to the surrounding genome. The number of regions from each region set that were covered by epigenetic data in the COCOA analysis is indicated by “*n*”

We also aimed to determine if COCOA could annotate inter-sample variation in multi-omics analyses that integrate epigenetic data with other data types. We therefore applied COCOA to a cohort of 200 chronic lymphocytic leukemia patients [22] with gene expression, ex vivo drug response, somatic mutation, and DNA methylation data. We used preprocessed data from MOFA (Multi-Omics Factor Analysis), a multi-omics dimensionality reduction method that summarized the high-dimensional data into 10 new dimensions referred to as latent factors (LFs) [23]. As part of the published analysis interpreting the 10 latent factors, the authors used a gene-centric method to annotate the latent factors with gene sets but only 5 could be associated with gene sets [10, 23]. Because COCOA works with data associated with genomic coordinates, we were able to use the DNA methylation data from the MOFA analysis with COCOA to annotate the latent factors with region sets. Since only a subset of the DNA methylation data was used for the MOFA calculations, we calculated the covariance of each CpG in the 450k microarray with each latent factor and used this matrix as input for COCOA. Using COCOA, we are able to annotate 4 of the 5 latent factors that were not associated with gene sets, demonstrating that COCOA’s region-centric approach complements the gene-centric approach applied by the MOFA authors (Fig. 4a). For latent factor 1 (LF1), we found variability in region sets for hematopoietic regulatory regions and transcription factors (Additional file 1: Table S3), consistent with the conclusions of the original paper that LF1 is related to the hematopoietic differentiation state of the leukemic cell of origin. The top region set for LF1 was enhancer regions in the GM12878 transformed B lymphocyte cell line, which had stark differences in DNA methylation across samples that correlated with IGHV mutation status, a marker of mature B cells that have undergone somatic hypermutation [24] (Fig. 4b). This result shows that COCOA was able to identify a plausible source underlying the latent factor, a result which was not identified using gene sets. As another example, we found region sets related to stem cell biology, including OCT4, NANOG, H3K4me1 from the H9 stem cell line, and SOX2, to be associated with LF8 (Fig. 4c, Additional file 1: Table S3). Since OCT4 and NANOG activity has been shown to be associated with β catenin [25–27], a mediator of WNT signaling, our results support and further expand upon the original association between LF8 and WNT reported by the MOFA authors. These results demonstrate that COCOA can enable richer multi-omics analysis by annotating the epigenetic component of inter-sample variation.

### COCOA reveals associations between epigenetic state and variation in sample phenotype

**Fig. 5:**
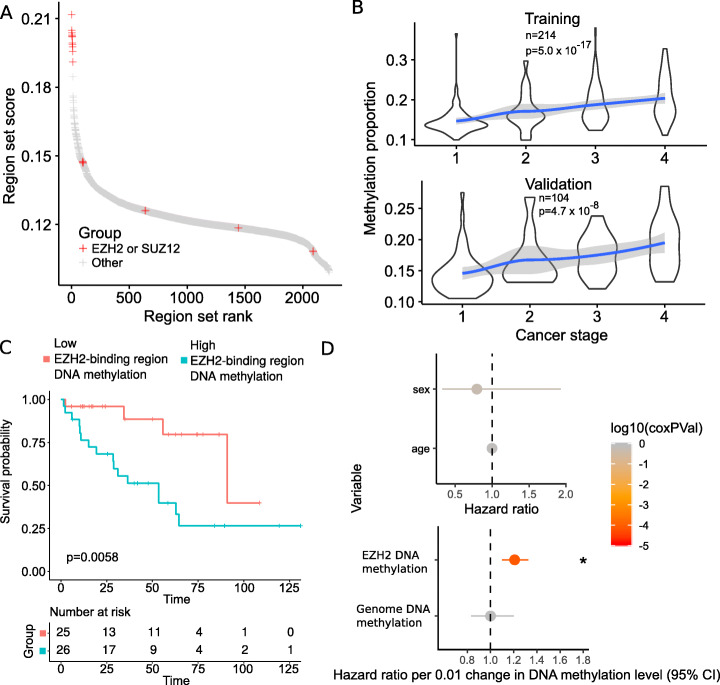
COCOA identifies region sets related to a patient phenotype of interest, cancer stage. **a** Region sets for the polycomb proteins EZH2 and SUZ12 were the top region sets related to cancer stage. **b** Average DNA methylation level in EZH2-binding regions (the top EZH2 region set) increases with cancer stage. *p* values by *t* approximation with null hypothesis that correlation is zero. **c** Kaplan-Meier curves of the validation samples, grouping samples by average DNA methylation in EZH2-binding regions (25% highest samples and the 25% lowest samples). *p* value from the log-rank test. **e** Cox proportional hazards model of average DNA methylation in the top EZH2-binding region set, correcting for age, gender, and average genome methylation level

The three examples thus far demonstrate how COCOA can be applied in an unsupervised analysis, which explores biological variation in the absence of known groups. To explore whether we could apply COCOA to a setting where groups or phenotypes are known, we extended COCOA to accommodate supervised analysis. For the supervised approach, we select a sample phenotype of interest (such as a molecular phenotype or a clinical outcome) and then measure the association of epigenetic variation with that parameter. To demonstrate a supervised COCOA analysis, we analyzed TCGA 450k methylation microarrays from kidney renal clear cell carcinoma (KIRC). This dataset includes a phenotypic annotation of cancer stage, which we used as our target variable. We hypothesized that COCOA could associate an epigenetic regulatory state with cancer stage and decreased survival. To test this hypothesis, we used COCOA to identify region sets where DNA methylation is correlated with cancer stage. We used a training-validation approach to assess the significance of our results (see the “Methods” section). In the training samples, COCOA identified polycomb protein (EZH2 and Suz12)-binding region sets to have the highest correlation with cancer stage (Fig. 5a, Additional file 1: Table S4). Next, we tested whether the average DNA methylation level in the top EZH2 region set is associated with cancer stage. In both training and validation samples, the average DNA methylation level in EZH2-binding regions had a significant positive correlation with cancer stage (*p* < 10^−16^ and *p* < 10^−7^, *t* approximation) showing that the COCOA result extends beyond the training set (Fig. 5b, Additional file 3: Table S5). Higher DNA methylation levels in EZH2-binding regions in advanced stages of cancer suggest that these regions could be repressed in advanced cancer stages, which would be consistent with higher activity of the repressive protein EZH2. This result is consistent with previous studies, which have found that higher EZH2 expression could promote metastasis in renal cell carcinoma [28] and other cancers [29–31] and is associated with a more advanced cancer stage [32, 33].

To further assess the relevance of our COCOA results, we tested the association between DNA methylation in our top EZH2 region set and patient survival. We compared the quartile of patients with highest average DNA methylation in the EZH2 region set to the quartile of patients with the lowest average DNA methylation, using a Kaplan-Meier estimate (Fig. 5c). Patients with higher EZH2 region set DNA methylation have significantly decreased survival compared to those with lower DNA methylation (*p* < 0.01, log-rank test, Fig. 5c). A Cox proportional hazards model correcting for age, gender, and average genome methylation levels also revealed a significant association between the average DNA methylation level in EZH2-binding regions and patient survival (*p* < 10^−4^, Fig. 5d, Additional file 4: Table S6). Previous studies found EZH2 expression to be prognostic for survival in renal cell carcinoma and other cancers [29, 32, 34], but to our knowledge, this is the first demonstration that DNA methylation levels in EZH2-binding regions could be prognostic for survival in renal cell carcinoma. We further assessed for cancer stage and survival association for the top TF region sets from the COCOA analysis. We tested the two highest scoring TFs—JUND and TCF7L2. In the validation data, DNA methylation in JUND-binding regions had a significant negative correlation with cancer stage (*p* = 0.022, *t* approximation) but we could not validate its association with survival because it did not satisfy the Cox proportional hazards assumption (Additional file 2: Fig. S7A, Additional file 4: Table S6). DNA methylation in TCF7L2-binding regions was not significantly correlated with cancer stage in the validation data (Additional file 2: Fig. S7B, Additional file 3: Table S5) but higher DNA methylation was significantly associated with better overall survival (*p* = 0.038, Cox proportional hazards model, Additional file 2: Fig. S7C, Additional file 4: Table S6). Through this supervised analysis, we demonstrate that COCOA can identify epigenetic variation related to a given sample phenotype of interest, providing a novel means for targeted analysis of epigenetic variation.

### DNA methylation in EZH2-binding regions is associated with cancer stage and survival in multiple cancers

**Fig. 6:**
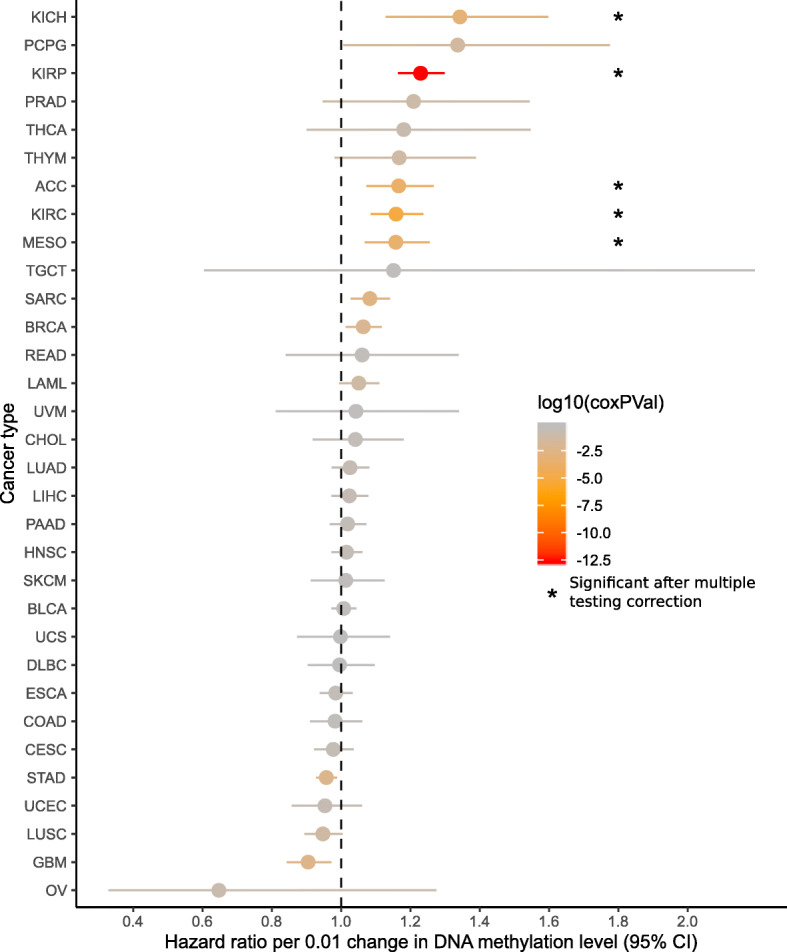
Pan-cancer survival analysis of DNA methylation in EZH2/SUZ12-binding regions. The mean hazard ratio and 95% confidence interval for the average DNA methylation in EZH2/SUZ12-binding regions are shown for each cancer type. Color indicates the raw *p* values and asterisks mark significance after Holm-Bonferroni correction

Given that COCOA identified associations with EZH2 region sets in both our unsupervised analysis of breast cancer and our supervised analysis of kidney renal cell carcinoma, we wondered whether the link with EZH2 and DNA methylation would hold true for other cancer types. To test this, we performed a pan-TCGA analysis investigating the association between average DNA methylation in EZH2/SUZ12-binding regions and cancer stage as well as overall patient survival. We combined regions from the top group of 11 EZH2 and SUZ12 region sets from the KIRC analysis (Fig. 5a, Additional file 1: Table S4) to generate a single EZH2/SUZ12 region set, referred to hereafter simply as EZH2-binding regions. We then computed the average DNA methylation in this region set for each sample and tested its association with either cancer stage or overall survival. We found a significant correlation between DNA methylation in EZH2-binding regions and cancer stage in multiple cancer types (Additional file 2: Fig. S8, Additional file 5: Table S7, Additional file 6: Table S8). DNA methylation in EZH2-binding regions positively correlated with cancer stage in 5 of 21 tested cancers, but trended negative in 3 cancer types (Additional file 2: Fig. S8), of which colon adenocarcinoma (COAD) had a significant negative correlation (*p* < 0.05, *t* approximation, Holm-Bonferroni correction), consistent with a previous report [35]. To further investigate the significance of the EZH2-binding regions, we used a Cox proportional hazards model to test for association between survival and average DNA methylation in these regions and found a significant association in 5 cancer types (Fig. 6, Additional file 6: Table S8, Additional file 7: Table S9). Similar to the cancer stage analysis, higher DNA methylation level was more often associated with increased risk of death, but trended to lower risk in a few cancer types (Fig. 6). This result is consistent with previous reports that EZH2 can be either oncogenic or a tumor suppressor [31, 36, 37] and emphasizes the context-specific effects of EZH2. Our pan-cancer analysis also supports previous reports suggesting that polycomb activity may be commonly dysregulated in cancer [31] and may influence survival in a variety of cancers, with some cancers having a positive and others a negative association [31]. Our results contrast with previous reports for several cancer types (Supplementary Discussion). This analysis identified a novel connection between EZH2 and survival in adrenocortical carcinoma (ACC), which has not been previously demonstrated. Furthermore, we have shown for the first time that variation in DNA methylation at EZH2-binding regions is associated with cancer stage and patient survival across a variety of cancers. Overall, this analysis demonstrates the ability of COCOA to annotate epigenetic variation and its potential to generate new mechanistic hypotheses about epigenetic heterogeneity and disease drivers.

### Comparison of COCOA to other methods

COCOA distinguishes itself from other methods by being the only method of its type for DNA methylation data and by its flexibility in supporting a wide range of analyses for epigenetic data. We conceptualize COCOA as being in a class of methods that relies on covariation of epigenetic signal to annotate epigenetic variation. This separates COCOA from the methods that annotate epigenetic variation without taking into account covariation. To demonstrate the power of this approach, we compared COCOA to LOLA, a method that does not consider covariation. This analysis demonstrated that COCOA has superior ability to mitigate noise (Supplemental information, Additional file 2: Fig. S9, Additional file 8: Table S10, Additional file 1: Tables S11 and S12). Other methods that do take into account covariation have key differences from COCOA. First, while tools exist that aggregate signal in related groups such as gene sets or region sets and use PCA to identify covariation of signal across samples (Table 1), no existing tool does this for DNA methylation data. Second, COCOA creates a generalized framework for region set analysis which results in great flexibility in applications. This generalized framework allows COCOA to be used in analyses that other tools may not support: with multiple epigenetic data types, for supervised or unsupervised analyses, with a variety of mathematical metrics, and for single-omic or multi-omic analyses. For a brief description of each method from Table 1 and further comparison to COCOA, see “Comparison of COCOA to other region set or covariation-based methods” in the supplementary text. Of the epigenetic tools with similar goals to COCOA, chromVAR [2] is the most widely used and most similar to COCOA in its input type. Therefore, we selected chromVAR for comparison to COCOA with the breast cancer ATAC-seq data. Each method revealed relevant but partially divergent aspects of inter-sample variation. COCOA had an improved ability to identify ER-related epigenetic variation and to separate biological signals with its use of PCA (Additional file 2: Fig. S10, Supplementary Information: “Comparison of COCOA to chromVAR”, Additional file 1: Table S2, Additional file 9: Table S13). COCOA also extends beyond chromVAR in COCOA’s analysis options and supported data types. COCOA thus provides a novel framework for flexible covariation-based analysis of DNA methylation and other epigenetic data.

**Table 1:**
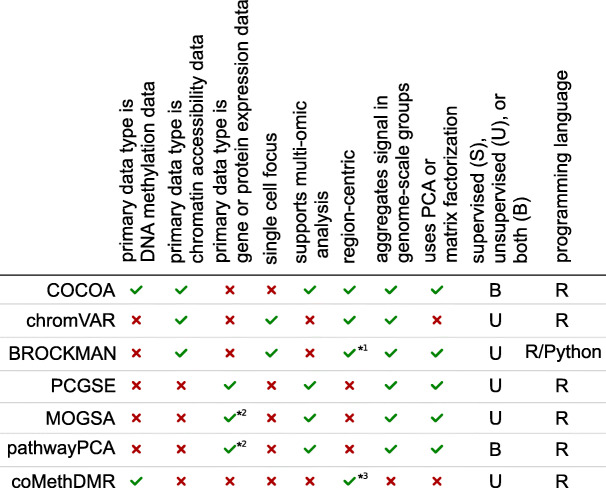
Features of COCOA and related methods

*^1^BROCKMAN uses k-mer counts, but the regions containing each k-mer can be conceptualized as a region set. *^2^MOGSA and pathwayPCA can involve multiple “omics” data types, but including gene-centric data such as gene or protein expression is important for the methods. *^3^coMethDMR finds differentially methylated regions but often annotates them in reference to genes

## Conclusion

We created a flexible framework for identifying and understanding sources of regulatory variation in epigenetic data. COCOA could be applied to any epigenetic data that has a value associated with genomic coordinates, which includes both nucleotide-level data such as bisulfite sequencing and region-based data such as ATAC-seq data. Our results also demonstrate how COCOA can be integrated with multi-omics analyses that include epigenetic data. Our tool allows scientists to leverage publicly available regulatory data to annotate variation in their epigenetic data. In an unsupervised analysis, COCOA can annotate the major axes of inter-sample variation. In a supervised analysis, COCOA can annotate inter-sample variation related to a specific phenotype of interest. We have released COCOA as a Bioconductor package [38], facilitating this new method of regulatory analysis. COCOA is a flexible and powerful method for interpreting regulatory variation between individuals.

## Methods

### COCOA algorithm

#### Overview

COCOA annotates variation in epigenetic data through two steps. In the first step, we quantify the association between each feature in the epigenetic data and the target variable using a metric such as correlation (Fig. 1e). This gives a score to each epigenetic feature that represents how much it is associated with the target variable. Then in the second step, we use the epigenetic feature scores to score region sets from a large collection of region sets (Fig. 1f). Finally, we use a permutation test to assess statistical significance, and return a ranked list of region sets.

#### Step 1: Quantifying variation across samples

COCOA starts with a data matrix of epigenetic signal values in genomic regions, where each row is a genomic locus (e.g., a CpG or an ATAC-seq region), and each column is a sample. The values in the matrix correspond to signal intensity levels (e.g., DNA methylation level or chromatin accessibility) of a given sample at a given locus. The first step in a COCOA analysis is to transform the original data into a score for each locus measuring how much it contributes to the target inter-sample variation. We refer to the score for an epigenetic feature (locus) as a “feature contribution score” (FCS). This calculation can be either supervised or unsupervised (Fig. 1c, d).

##### Supervised

For supervised analyses, the goal is to identify sources of variation associated with a target sample phenotype of interest. Therefore, in addition to the epigenetic data matrix, we require a vector representing the target sample phenotype. We then quantify the association between the target sample phenotype and the epigenetic signal at each genomic locus using a method such as Pearson correlation. We end up with a vector of scores (which for correlation is the correlation coefficient) representing how strongly epigenetic variation at a genomic locus is associated with variation in the sample phenotype. Metrics other than Pearson correlation can be used to quantify variation, as long as they produce a score for each genomic locus. A detailed discussion of metric choice follows in the section “Metric for quantifying variation.”

##### Unsupervised

For unsupervised analyses, we first apply a dimensionality reduction technique such as PCA or MOFA [23] to identify latent factors that represent significant sources of inter-sample variation [10]. Then, we treat these latent factors as target sample phenotypes and quantify the association between each latent factor and the epigenetic data as we would for the sample phenotype in the supervised analysis. In this case, the feature contribution score for each genomic locus represents how strongly epigenetic variation at that genomic locus is associated with variation in the latent factor.

#### Step 2: Annotate variation with the COCOA algorithm

After quantifying inter-sample variation, we are left with one or more vectors that assign FCS to each genomic locus in the original data matrix. COCOA next seeks to determine which region sets are associated with that variation. For this step, COCOA relies on a database of region sets. Here, we have used a subset of the LOLA database [1], which includes several thousand region sets that have been manually collected from several large-scale experiments and databases, including the ENCODE [39, 40] and Roadmap Epigenomics projects [41, 42]. For the sample-specific data, COCOA can operate on two types of signal data: single-nucleotide data (e.g., DNA methylation) or region-based data (e.g., ATAC-seq peaks). In either case, we will aggregate the scores for all individual genomic loci into a combined score for each region set (Fig. 1d). Due to different experiments testing the same TF or histone modification, some region sets share similar regions to each other and therefore their scores are not completely independent.

For single base-pair resolution data (e.g., DNA methylation data), the following algorithm is used for a single region set and a single FCS vector: First, we optionally take the absolute value of the FCS (Supplementary Methods). Then, we identify all features whose genomic coordinates overlap the given region set. Within each region from the region set, we average the FCS of any overlapping features to get a single average value for each region. We then average the region scores to get the final score for that combination of region set and FCS vector. This score represents how much that region set is associated with the latent factor or phenotype that corresponds to the FCS vector. We repeat this process for each pairwise combination of region set and latent factor/phenotype FCS vector.

For region-based data such as ATAC-seq data, the scoring is conceptually similar to single-nucleotide data, but with slight differences. We use the following algorithm: To score a region set for a given latent factor or phenotype, we first identify all overlaps between “data regions” (regions for the epigenetic signal data) and region set regions. For each overlap, we calculate what proportion of the region set region is overlapped by the data region. We then take a weighted average of the FCS of all the overlapping data regions, weighting each data region’s FCS by the proportion that region overlaps a region set region and dividing by the sum of all overlap proportions. This weighted average is the region set score that represents how much the region set is associated with the latent factor or phenotype. We repeat this process for each combination of region set and latent factor/phenotype.

COCOA also offers alternative scoring methods including the option to use the median instead of the mean. We discuss this option in the Supplementary Discussion where we compare results for COCOA of breast cancer DNA methylation using median and mean scoring methods, finding overall similar results and high correlation between median and mean scores (Additional file 2: Fig. S11, Additional file 1: Tables S1, S14). Other scoring options can be found in the software documentation.

#### Metric for quantifying variation

Choosing an appropriate metric can help to effectively capture the relationship between epigenetic variation and variation in the target variable (Supplementary Methods). In this paper, we used covariance, Pearson correlation, Spearman correlation, PCA, and MOFA [23] to quantify variation, but other variation metrics and dimensionality reduction techniques can be used with COCOA for quantifying inter-sample variation, depending on the specific circumstances of a given analysis. The only requirement is that the metric must provide a score for each epigenetic locus that quantifies how much it is associated with variation in the target variable. The choice of metric can depend on the data type.

For DNA methylation data, since DNA methylation data is bounded from 0 to 1, we used covariance to give greater weight to CpGs with larger changes in DNA methylation across samples. Since the range of ATAC-seq counts could be very different between different peaks, we used Pearson correlation for the ATAC-seq data in order to give each peak a comparable score, regardless of the peak’s range. This principle also applies to PCA. When performing PCA, we recommend scaling the data by dividing each variable by its variance for ATAC-seq data (equivalent to correlation) but not for DNA methylation data (equivalent to covariation). Then, when treating the principal components as the target variables, we use the corresponding metric—covariance or correlation—to get the feature scores. We recommend Spearman correlation when the relationships between the target variable and the epigenetic features are monotonic but not linear, as may occur when the target variable is ordinal (e.g., cancer stage).

### Permutation test

To assess the statistical significance of the COCOA results, we use a permutation test. For both supervised and unsupervised COCOA analyses, we have a target variable (i.e., the sample phenotype or latent factor) and want to understand the relationship between the target variable and the epigenetic data. For a single permutation, we randomly shuffle the samples’ target variable values then recalculate the association between the epigenetic data and the target variable as done in step 1 (Fig. 1e). This gives each epigenetic feature an FCS for the shuffled target variable. Then, we run COCOA on the new feature contribution scores to score each region set in the database. This process is repeated for each permutation. The COCOA scores for a given region set from the permutations form a region set-specific null distribution. Because the sample labels were shuffled instead of the epigenetic data, the null distributions can appropriately capture the correlation structure of the epigenetic data, accounting for the correlation between epigenetic features in a given region set. The region set-specific null distributions also protect against false positives that could arise from some region sets being more fully covered by the epigenetic assay than others because each score in a region set’s null distribution is created from the same coverage profile. To reduce the computational burden, we calculated 300 permutations and applied a permutation approximation technique [43]. We fit a gamma distribution to each null distribution using the method of moments in the fitdistrplus R package [44] and then calculated a *p* value for each region set using its gamma distribution. To test the appropriateness of fit of the gamma approximation, we ran a simulation study with 100,000 permutations and then subsampled and applied the approximation to see how close the approximation is to the true *p* value. Our conclusion is that the gamma approximation is accurate for high *p* values, but the gamma approximation may overestimate the significance of low *p* values; therefore, we advise that it can be helpful for screening out region sets that are not significant (Fig. S12; further discussion in Supplementary Information). To correct *p* values for the number of region sets tested, we used Benjamini-Hochberg false discovery rate (FDR) correction [45] with an FDR of 5%.

### Meta-region profile plots

To visualize results, COCOA produces a plot we call the *meta-region profile* plot (e.g., Figure 2c). The goal of the meta-region profile is to compare the feature contribution scores in the regions of interest to the surrounding genome to assess how specific the captured signal is to a region set. We combine information from all regions of the region set into a single summary profile as has been done for DNA methylation data [3, 6]. Each region in the region set is expanded on both sides to include the surrounding genome (e.g., expanded to 14 kb total, centered on the region of interest). This enlarged region is then split into bins of approximately equal size. Finally, the FCS for corresponding bins from each region of the region set are averaged to get a single “meta-region” FCS profile. By default, the type of average depends on whether the data is single base-pair resolution or region-based, with the same algorithm applied as for the COCOA score. A peak in the middle of the profile suggests that there is variation that is specific to this region set.

### Region set database

To annotate variation in the epigenetic data, we used a subset of the LOLA database [1] (filtered with R script, see Supplementary Materials), totaling 2246 region sets from public sources. Sources included the ENCODE project [39, 40], Roadmap Epigenomics [41, 42], CODEX database [46], and the Cistrome database [47]. Additionally, we included some region sets derived from JASPAR motif [48] predictions. Examples of region sets include transcription factor-binding sites from ChIP-seq experiments, histone modification regions from ChIP-seq experiments, and cell type or condition-specific accessible chromatin from ATAC-seq experiments. For a discussion of how to choose a region set database and other related considerations, see “Additional file 2”. For each analysis, we only considered in the results region sets that had at least 100 regions with any coverage by the epigenetic data. Since the CLL MOFA data was in reference genome hg19 and the breast cancer data was in hg38, we used the corresponding hg19 or hg38 version of the region set database when analyzing each dataset. A brief description of the region sets can be found in the supplementary data (Additional file 1: Tables S1-S4), and the database is available at http://databio.org/regiondb [1]. To designate region sets “hematopoietic TFs” for Fig. 3, we did a literature search, selecting three reviews: one focusing on myeloid TFs [49], one focusing on lymphoid TFs [50], and one general hematopoietic TF [51]. The hematopoietic TFs identified from these reviews are the following: RUNX1, TAL1, PU.1, CEBPA, IRF8, GFI1, CEBPE [49], TCF3, EBF1, PAX5, FOXO1, ID2, GATA3 [50], KLF1, GATA1, GATA2, IKZF1, CMYB, and NFE2 [51]. Since GATA3 was also identified as an ER-related TF, we did not consider GATA3 as a hematopoietic TF in plots to avoid confusion.

### Breast cancer analyses

#### Datasets

For the unsupervised breast cancer analyses, we used DNA methylation and ATAC-seq datasets from The Cancer Genome Atlas (TCGA). We retrieved the DNA methylation and clinical data with the TCGAbiolinks R package [52]. We identified 657 patients with both 450k DNA methylation data and known ER and progesterone status. For the ATAC-seq data, we retrieved a peak count matrix for the consensus set of breast cancer ATAC-seq peaks identified by Corces et al. from the following location: https://atacseq.xenahubs.net/download/brca/brca_peak_Log2Counts_dedup. We used a sample ID lookup table to match the ATAC-seq IDs to the standard TCGA identifiers: https://gdc.cancer.gov/about-data/publications/ATACseq-AWG. We excluded one patient of the 74 patients with ATAC-seq data (TCGA-AO-A0J5) for whom we did not have sufficient metadata.

#### Data processing and quantifying variation

For the breast cancer DNA methylation data, we excluded the sex chromosomes. For the ATAC-seq data, we used the peak count matrix from Corces et al. [21], without further processing. We performed PCA on the DNA methylation data and the ATAC-seq data separately with the “prcomp” R function, with centering and without scaling. PCA is used to get covariance of features and to prioritize the largest sources of covariance. After PCA, we calculated the covariance or correlation coefficient for each epigenetic feature with each latent factor to get a value that represented how much each feature contributed to each latent factor. We used covariation for the DNA methylation data and correlation for the chromatin accessibility data. To test the association of ER status with PC score, we used the Wilcoxon rank-sum test with ER-positive samples and ER-negative samples as the two groups.

#### Comparison of COCOA and chromVAR

To compare COCOA and chromVAR [2], we completed two tests with the breast cancer ATAC-seq data. First, we applied chromVAR with the same region set database used by COCOA in our ATAC-seq analysis. Second, we applied COCOA and chromVAR with the main motif database used by chromVAR in its publication, which is a curated version of the cisBP database [53] and is available as the “human_pwms_v1” data object from the “chromVARmotifs” R package that can be downloaded from the “GreenleafLab/chromVARmotifs” Github repository. We applied chromVAR to the normalized data from Corces et al., adding a pseudocount to bring the minimum normalized signal up to zero. To use the motif database with COCOA, we identified peaks with motif hits using the “matchMotifs” function from the “motifmatchr” R package [54] with default parameters and took those regions as a region set. The “matchMotifs” function is the method chosen by chromVAR authors for identifying motif matches in the chromVAR Bioconductor vignette. For the chromVAR figure, we designated motifs as AP-1-related based on an AP-1 review (Figure 1 of review) [55].

### Multi-omics chronic lymphocytic leukemia analysis

#### Datasets

For the unsupervised multi-omics analysis, we used preprocessed data that was included with the MOFA R package, specifically the latent factors from the multi-omics dimensionality reduction analysis of 200 chronic lymphocytic leukemia (CLL) patients as described by Argelaguet et al. [22, 23]. We retrieved the 450k DNA methylation data for these patients using the ExperimentHub R package [56] (CLLmethylation data package, ExperimentHub ID: EH1071) [22].

#### Data processing and quantifying variation

For the multi-omics analysis, we used the dimensionality reduction results from the paper by Argelaguet et al. and then extended the results to CpGs that were not included in the dimensionality reduction. The original multi-omics analysis used only the most variable 1% of CpGs (4248 CpGs) for calculation of the latent factors. Since COCOA benefits from higher coverage of CpGs across the genome, we calculated the correlation of each CpG from the DNA methylation microarrays (excluding sex chromosomes) with each latent factor. This yielded a matrix with CpG, latent factor correlations where each row is a CpG and each column is a latent factor, which can be used as input to COCOA.

### Kidney renal clear cell carcinoma analysis

#### Dataset

For the supervised KIRC analysis, we used DNA methylation and clinical data from The Cancer Genome Atlas. We used 450k DNA methylation microarray data for 318 patients, retrieved with the curatedTCGAData R package [57]. The clinical data included cancer stage and survival information that was used to label samples in the supervised analysis.

#### Data processing and quantifying variation

For the supervised analysis of KIRC methylation, we first split the data into two groups: training (2/3 of patients) and validation (1/3 of patients), keeping approximately equal proportions of each cancer stage in each group. With the COCOA samples, we first calculated the Spearman correlation between the DNA methylation levels and the sample phenotype of interest, cancer stage. This resulted in a correlation coefficient for each CpG. We then applied the COCOA algorithm on the absolute correlation coefficients.

#### Validation and survival analysis

After running COCOA on 2/3 of the samples, we did validation analyses on the remaining 1/3 of samples. First, we tested whether each patient’s average DNA methylation level in the top EZH2 region set from COCOA was correlated with cancer stage, using the “cor.test” R function [58] and Spearman correlation. To calculate correlation *p* values for the null hypothesis that the correlation was zero, we used an asymptotic *t* approximation, the default method used by the “cor.test” function. To calculate the average methylation, we first separately averaged DNA methylation within each EZH2 region, then averaged all the region averages. We also tested whether average DNA methylation in EZH2 regions was related to overall patient survival. We created Kaplan-Meier curves with two groups: the 25% of validation samples with highest DNA methylation in EZH2 regions and the 25% of samples with the lowest DNA methylation. We used a log-rank test from the “survminer” R package’s “ggsurvplot” function [59] to get a *p* value for the Kaplan-Meier curves. We created a Cox proportional hazards model with all validation samples, relating average DNA methylation in EZH2 regions to patient survival and correcting for age, gender, and average genome methylation level. We also tested the two highest scoring TF region sets from the COCOA analysis—JUND and TCF7L2—for association with cancer stage and survival using the methods described above. We tested whether variables satisfied the proportional hazards assumption using the “cox.zph” function in R [60–62] (Additional file 4: Table S6), considering variables with *p* < 0.05 as not satisfying the assumption. The JUND validation model did not meet the assumption for the variable of interest (average DNA methylation in EZH2/SUZ12-binding regions) and therefore was not considered.

### Pan-cancer EZH2 analysis

In this analysis, we tested whether the average DNA methylation level in EZH2-binding regions would be associated with cancer stage and patient survival in other cancer types than KIRC. We combined regions from the top group of 11 EZH2 and SUZ12 region sets from the KIRC analysis (Fig. 5a, Supplementary Data) to make a single “master” EZH2/SUZ12 region set (referred to as EZH2-binding regions). We took the union of all regions and merged regions that overlapped. We downloaded DNA methylation microarray data for 33 TCGA cancer types using the curatedTCGAData R package [57]. Then, for each sample, we calculated the average DNA methylation level in EZH2-binding regions. For each cancer type for which we had cancer stage information (21/33), we calculated the Spearman correlation between average EZH2-binding region DNA methylation and cancer stage, using the “cor.test” R function [58]. To calculate correlation *p* values for the null hypothesis that the correlation was zero, we used an asymptotic *t* approximation, the default method used by the “cor.test” function. Next, for each cancer type, we used a Cox proportional hazards model to test the association of average EZH2-binding region DNA methylation with survival, with the covariates patient age, sex, and average microarray-wide DNA methylation level as available. We tested whether variables satisfied the proportional hazards assumption using the “cox.zph” function in R [60–62] (Additional file 7: Table S9). We considered variables with *p* < 0.01 as not satisfying the assumption, picking a more stringent cutoff because more models were tested. Models that did not meet the assumption for the variable of interest (average DNA methylation in EZH2/SUZ12-binding regions) were removed, in our case only one cancer type—low-grade glioma (LGG). We corrected Spearman and Cox *p* values for multiple testing using the Holm-Bonferroni method [63].

## Supplementary information

**Additional file 1: Tables S1-S4, Tables S11-S12, Tables S14-S15.** COCOA region set scores for the corresponding analysis. **Table S1.** BRCA DNA methylation data. **Table S2.** BRCA ATAC-seq data. **Table S3.** CLL multi-omics analysis. **Table S4.** KIRC cancer stage analysis. **Table S11.** Results for simulated DNA methylation data with low noise level. **Table S12.** Results for simulated DNA methylation data with high noise level. **Table S14.** BRCA DNA methylation data with median scoring method, **Table S15.** BRCA DNA methylation data when using loadings as FCSs.

**Additional file 2: Supplemental figures S1-S12** supplemental methods, and supplemental discussion.

**Additional file 3: Table S5.** Spearman correlation between average DNA methylation in each region set and KIRC cancer stage for training and validation data.

**Additional file 4: Table S6.** Results from Cox proportional hazards model for the three training models and three validation models that tested the relationship of average DNA methylation in each region set with overall patient survival.

**Additional file 5: Table S7.** Spearman correlation between average DNA methylation in EZH2/SUZ12 region set and cancer stage for each cancer type.

**Additional file 6: Table S8.** Association between average DNA methylation level in EZH2/SUZ12-binding regions and cancer stage or patient survival.

**Additional file 7: Table S9.** Results from Cox proportional hazards model for each cancer type that tested the relationship of average DNA methylation in the EZH2/SUZ12 region set with overall patient survival.

**Additional file 8: Table S10.** LOLA results from differentially methylated regions from simulated DNA methylation data with low noise.

**Additional file 9: Table S13.**. COCOA and chromVAR results for breast cancer ATAC-seq data using chromVAR motif database.

**Additional file 10.** Review history.

## Data Availability

The R scripts used for this analysis can be accessed at https://github.com/databio/COCOA_paper [64]. The COCOA package can be accessed at http://bioconductor.org/packages/COCOA [65]. An archived version of both is also available [66] (DOI: 10.5281/ZENODO.3973375). These are released under the open source BSD-3-Clause license. We retrieved the BRCA DNA methylation and clinical data with the TCGAbiolinks R package [52]. We retrieved the KIRC DNA methylation and clinical data with the curatedTCGAData R package [57]. For the BRCA ATAC-seq data, we used the peak count matrix from Corces et al. [21], which we retrieved from https://atacseq.xenahubs.net/download/brca/brca_peak_Log2Counts_dedup. We used a sample ID lookup table to match the ATAC-seq IDs to the standard TCGA identifiers: https://gdc.cancer.gov/about-data/publications/ATACseq-AWG. For the CLL multi-omics analysis, we retrieved the 450k DNA methylation data for these patients [22] using the ExperimentHub R package [56] (CLLmethylation data package, ExperimentHub ID: EH1071). We retrieved the MOFA latent factors and patients’ mutation info from the MOFAdata R package [67]: https://bioconductor.org/packages/release/data/experiment/html/MOFAdata.html To create hematopoietic chromatin accessibility region sets, we retrieved an ATAC-seq count matrix (GSE74912_ATACseq_All_Counts.txt.gz) from Gene Expression Omnibus with hematopoietic ATAC-seq data from Corces et al. [68], which was further processed as described in the “Methods” section. The remaining region set database can be downloaded at http://databio.org/regiondb. The chromVAR motif database was retrieved from https://github.com/GreenleafLab/chromVARmotifs [69].
