# Supplementary material for: COCOA: coordinate covariation analysis of epigenetic heterogeneity
Source: Genome Biol. 2020 Sep 7;21:240. doi: 10.1186/s13059-020-02139-4 (PMC7487606; doi:10.1186/s13059-020-02139-4)
Supplement: Supplementary file 2 — Additional file 2: Supplemental figures S1-S12 supplemental methods, and supplemental discussion. [file 13059_2020_2139_MOESM2_ESM.docx]

**Supplementary Methods and Information for COCOA**

**The power of covariation in analysis of epigenetic heterogeneity**

Covariation of the epigenetic signal in different regions is an important principle in epigenetic analysis but is not fully taken advantage of by many epigenetic analysis methods. There are two common limitations of analysis methods. First, relying on differential signals between discrete sample groups loses information about the differences among samples within a group. For instance, in a health-related differential analysis, patients in the “disease” group are considered equal for the analysis when there may actually be differences between patients in the severity of their disease. Although some variation can be effectively summarized by discrete groups, in some cases, it is often more appropriate to consider variation along a continuous spectrum^[70]^. Using a continuous spectrum for samples based on physical or molecular phenotype instead of discrete groups could provide greater resolution for identifying epigenetic features that covary with sample status. Second, the use of discrete cutoffs for identifying significant epigenetic differences between samples loses information about the strength of covariation between epigenetic features and sample status. For example, epigenetic differences between samples are often determined using a discrete threshold that places epigenetic features into two groups – significantly different or not significantly different – as is done when finding differentially methylated or differentially accessible regions. Then the significant regions can be annotated with reference region sets through region set enrichment testing to aid interpretation^[1, 8, 71-74]^. However, while this is a flexible approach, converting continuous epigenetic signals to a binary classification -- significant or not significant -- results in the loss of covariation information that could be valuable for the region set enrichment analysis. This choice is a trade-off between the computational efficiency that comes from a simplified representation of the epigenetic signal and the potential gains that could come from having higher resolution data and most region set enrichment tools choose the simpler approach.

**Selection of immune cell-specific ATAC-seq region sets**

We retrieved an ATAC-seq count matrix (GSE74912_ATACseq_All_Counts.txt.gz) from Gene Expression Omnibus with hematopoietic ATAC-seq data from Corces et al.^[68]^. We normalized each sample with quantile normalization first then GC normalization with the cqn R package^[75]^, according to the normalization done by Corces et al.^[68]^. When there were multiple samples of a given cell type from a single individual, we calculated the mean of each region to combine them into a consensus count vector. From the counts for a given cell type from various individuals, we calculated the mean in each region to create a consensus set of counts for that cell type. To get custom hematopoietic region sets, we did a series of comparisons between cell type count profiles to determine regions that were open in one or a specific group of cell types and closed in another cell type or group of cell types. We counted regions as specific when they were in the top 10% of regions in the chosen cell type/s and in the bottom 50% of the other compared cell type/s. The code for creating these region sets is available in the 0-ClusterHemaATAC.R file.

**Creation of simulated data**

To create simulated data to test COCOA, we first calculated an aggregate healthy DNA methylation profile by averaging the DNA methylation profiles of 160 TCGA healthy kidney samples. To get a true positive region set, we selected an arbitrary region set (ER) and set the DNA methylation of all CpGs that overlapped that region set to zero in the healthy sample. For our analysis, we used 10 replicates of the healthy sample. We created 10 artificial disease samples by changing the DNA methylation of the CpGs in the region set of interest to between 0.0125 and 0.25, depending on the sample, with all CpGs in a given sample being assigned the same DNA methylation level. This results in covariation of the DNA methylation level of CpGs in the region of interest across samples and in differential methylation between healthy and disease samples. Finally, we added Gaussian noise to each CpG for each sample to create variation between samples, keeping methylation in the 0-1 range. We created two sample sets with different noise levels: low noise (mu=0, sd=0.025) and high noise (mu=0, sd=0.05).

To create region sets with a range of p-values, we made a set of region sets that had varied proportions of true positive regions and random loci sampled from the simulated data DNA methylation coordinates. Each random locus was expanded from the center to be 500 bp. To assign p-values to the region sets, we performed PCA on the high noise simulated data then ran COCOA on PC1 and PC2 with our region sets as the region set database. We calculated 100,000 permutations to determine empirical p-values for each region set. For further analysis in gamma approximation simulations, we selected region sets with empirical p-values across a range of orders of magnitude.

**How to choose a method for quantifying variation**

The choice of method for quantifying variation depends on the data and how well that method can prioritize features that covary with each other or with a sample phenotype of interest. The decision to use covariation or correlation depends on whether the epigenetic data is proportion-based, such as for bisulfite sequencing, or count-based and unbounded, such as for ATAC-seq. This decision is not expected to greatly affect the analysis but using correlation might give greater weight to epigenetic features with very small absolute changes across samples that actually represent noise and not real signal. Using covariation may be better for proportion-based data, such as for bisulfite sequencing, and correlation may be better for count-based data, such as for ATAC-seq. Since the concept of COCOA is based on the covariation of epigenetic features across samples, COCOA will work best with methods that prioritize covarying/correlated features and do not give lower scores or coefficients to correlated features. For instance, a simple regression gives coefficients to input variables based on their association with a dependent variable. However, if two input variables are correlated, regression will give a lower coefficient to one of two variables. PCA, on the other hand, can give a high loading value to both correlated variables. An assumption of our method is that a single regulatory signal will be related to multiple regions that are regulated in a coordinated way and therefore covary across samples. For example, we would expect that the epigenetic signal in cell type-specific regions would covary across samples depending on how much of each sample corresponded to that cell type. Therefore, we expect that COCOA would work best with methods that do not lower the coefficients or scores of variables that covary. While we generally used linear metrics for quantifying variation in this study, we expect that nonlinear metrics such as feature importance scores from machine learning models would also work for quantifying epigenetic variation if they meet the criteria described above.

Some readers may notice that we use covariation or correlation instead of simply using the PCA loadings as feature contribution scores for unsupervised COCOA. Since the principal component loadings also represent the contribution of each feature to the respective principal component, we could have used those as the feature scores. However, this would have required us to recompute the PCA for each COCOA permutation to get new loadings. Instead, for each permutation, we shuffle the PC scores and calculate the covariance or correlation between the shuffled PC scores and the epigenetic data. This allows us to get new feature scores for each permutation without recalculating the PCA for every permutation, which would be computationally expensive.

**Gamma distribution p-value approximation**

We used simulated data to compare empirical p-values from COCOA to the p-values derived from a gamma approximation. As mentioned earlier, we created simulated DNA methylation data with variation in the regions of a specific region set. We also created a collection of region sets that had varied similarity to the true positive region set and calculated their empirical p-values with 100,000 permutations of COCOA. To evaluate the accuracy of the gamma distribution approximation, we subsampled from the 100,000 permutations and used the subsampled COCOA runs to create gamma p-values. We did this for three subsample sizes: 300, 1000, and 10,000. For each subsample size, we sampled 500,000 times, calculating the gamma p-values each time. As seen in Fig. S12, the median gamma p-value is fairly close for high p-values but tends to be lower than the empirical p-values as the p-value decreases. Increasing the number of permutations from 300 to 10,000 reduced the variance of the gamma p-values but did not cause them to converge to the empirical p-values (Fig. S12). Because of this, we recommend caution when interpreting gamma p-values, with the reminder that it is an approximation. The main benefit of the gamma p-value approximation is to screen out region sets that are not significant, which are the region sets whose p-values fall in the range where the gamma p-value approximation is more accurate.

**Considerations when choosing a region set database**

The choice of region set database depends partially on the goals of the analysis but a broad database with region sets from a variety of transcription factors and cell types should be sufficient for most exploratory analyses. Along those lines, the region sets we used from ENCODE, Roadmap Epigenomics, and other sources provide a reasonably broad sampling of transcription factors and histone modification regions for a variety of cell lines and tissue types. However, any similar source of region sets could be used. The curation of region set databases is an active research area. Additionally, new region sets are continually being made available to the public. The user would benefit from any source of region sets that is relevant to their experimental question. This includes region sets derived from a cell or tissue type that is similar to the samples being studied, especially because transcription factor binding and many epigenetic marks including DNA methylation can be cell type-specific. If the user is asking a very targeted question about a specific transcription factor or cell type, the user may want to find a published region set through a source such as the Gene Expression Omnibus and use that region set alongside a broader region set database. While the database we used is not comprehensive, it is a rich starting point that can be expanded in the future.

**Other COCOA parameters**

***Absolute value of FCS***

After generating the feature contribution scores (FCS), the COCOA user has the option of taking the absolute value of those scores before scoring the region sets. This choice depends on whether all regions in a region set are expected to be regulated in the same way or not (i.e. all regions activated/all regions repressed or some regions activated and some regions repressed). For cases where regions in a region set are regulated in the same direction (all activated or all repressed), it would be better to not take the absolute value. Since the FCS for important regions should all have the same sign in this case, the relevant signal will be preserved during the COCOA aggregation step while the noise from irrelevant epigenetic features, which should have arbitrary FCS signs, will cancel out. For example, a TF might activate all regions where it binds and we would expect that the epigenetic signal in these regions would generally change in the same direction and have FCS with the same sign. For cases where regions in a region set are regulated in opposite directions (some activated and some repressed), the absolute value should be taken. Since the relevant signal may have some positive and some negative FCS, aggregating FCS without taking the absolute value would partially cancel out and diminish the signal. For example, a TF might activate some regions but repress others depending on what other proteins are binding with it at a given region. In this case, the epigenetic signal in regions bound by the TF might change in opposite directions, leading to FCS with opposite signs. When taking the absolute value, it is still possible to identify region sets where regions all change in the same direction. However, FCS that represent noise will not cancel out, potentially reducing the ability to discriminate between true signal and noise. In this study, we took the absolute value of the FCS when running COCOA since there may have been some region sets in our database with regions that are regulated in opposite directions.

***Scoring based on mean versus median***

COCOA offers the option to score based on the median region set FCS instead of the mean FCS. To compare the median scoring method to the mean scoring method which was used in the main text, we performed COCOA with the median scoring method on the TCGA breast cancer DNA methylation data. We see that the overall trends are similar, with ER-related region sets found to be highly ranked for PC1 and PC3 and polycomb-related region sets highly ranked for PC4 (Fig. S11A, Additional File 1: Table S14). Additionally, the meta-region profiles for top region sets from the mean scoring method also have peaks for the median scoring method (Fig. S11B). Consistent with these observations, the region set scores for the first 4 PCs have very high Spearman correlation between scoring methods, all with at least 0.95 correlation (Fig. S11C).

**Discussion of EZH2 results in comparison to previous findings**

Several trends present in our EZH2/SUZ12-binding region analysis contrast with previous results. First, we found a significant positive correlation between EZH2-binding region DNA methylation and cancer stage in testicular germ cell tumors (TGCT), whereas previous studies did not identify an association between EZH2 expression and cancer stage^[76]^ and suggested that EZH2 activity is decreased during cancer progression^[76]^ and in chemotherapy resistance^[77]^. Second, we found a negative correlation between EZH2-binding region methylation and cancer stage in UVM that trended toward significance (uncorrected p<0.05) while a previous study suggested that increased expression of EZH2 was positively associated with higher risk of metastasis^[30]^. Third, our finding that higher DNA methylation in EZH2-binding regions trended toward significance (uncorrected p<0.05) for association with lower risk of death in GBM contrasts with reports suggesting that EZH2 expression promotes proliferation and tumorigenesis in glioblastoma^[78, 79]^. These trends could be due in part to the context-dependent effects of EZH2^[31, 36, 37]^. Further studies would be valuable to clarify the role of EZH2 in these cancer types.

**Comparison of COCOA to other region set or covariation-based methods**

We are not aware of any other tool designed for DNA methylation data that identifies region sets based on DNA methylation variation across samples. However, since COCOA is broadly applicable to epigenetic data, we provide a comparison between COCOA and tools designed for chromatin accessibility data with which COCOA shares some important concepts. We also compare COCOA to tools that were not designed for epigenetic data but have some conceptual similarity to COCOA. Finally, we mention a tool designed for DNA methylation data that has superficial similarity to COCOA but actually performs a very different function. COCOA is unique in that it provides a class of DNA methylation heterogeneity analysis that was not previously available but also provides a framework to apply the same method to other epigenetic data types.

***Tools for chromatin accessibility data***

***ChromVAR.*** ChromVAR is an R package that quantifies the variability of chromatin accessibility signal in motif regions or region sets^[2]^. For a given set of motif regions, each sample is given a score for how much it deviates from the expected chromatin accessibility of those motif regions. Each motif region set is also given a score for how variable it is across samples. ChromVAR has a few major differences from COCOA. First, as mentioned previously, COCOA works with DNA methylation data or chromatin accessibility data, while chromVAR was designed for chromatin accessibility data. Second, COCOA can use multiple metrics to quantify epigenetic variation across samples while chromVAR only uses a single unsupervised way of quantifying variation (bias-corrected z-score for each sample, region set combination). Among COCOA’s multiple options, COCOA can use PCA to more easily separate and annotate biological signals. COCOA also supports supervised analysis, adding the ability to do a range of new analyses not supported by chromVAR. Third, a smaller point, COCOA includes additional data analysis and visualization functions such as for meta-region profiles to further understand inter-sample variation. While chromVAR’s utility is attested to by the many papers citing it, COCOA adds meaningful value to the epigenetics field that is not captured by the chromVAR package.

***BROCKMAN.*** BROCKMAN is a tool designed primarily for single cell chromatin accessibility data that uses variation in the frequency of k-mers in reads to identify gene regulatory variation across cells^[9]^. While BROCKMAN and COCOA share some conceptual foundations, specifically that covariation of regulatory signals across cells or samples can be used to understand gene regulatory differences between the cells, there are some important differences. First, the BROCKMAN tool is for chromatin accessibility data, not DNA methylation, while COCOA has a generalized framework that works for both data types. Second, BROCKMAN aggregates epigenetic signal by category (k-mer) before doing dimensionality reduction while COCOA first does dimensionality reduction (or other quantification method) then aggregates epigenetic signal by category (region set). Aggregating before dimensionality reduction is well suited to single cell data, as has been done for single cell DNA methylation data ^[80]^. However, aggregating epigenetic signal after dimensionality reduction allows more flexibility in applications and allows genome-wide variability to be captured in a more unbiased way. For example, COCOA could be used with multi-omic dimensionality reduction as shown in Figure 4 with minimal changes to the COCOA algorithm. Aggregating signal within region sets first might miss inter-sample epigenetic variability that is not contained within any tested region sets. COCOA shares some ideas with BROCKMAN but applies them in a generalized framework that can apply to new epigenetic data types, including DNA methylation.

***Gene-centric methods with conceptual similarity to COCOA***

The next three methods have some conceptual overlap with COCOA but are gene-centric rather than region-centric. As mentioned in the paper introduction, region-based approaches are more appropriate for epigenetic data, for reasons including that it can be difficult to link epigenetic marks to genes.

***PCGSE.*** Principal component gene set enrichment (PCGSE) is a method to annotate principal components that are derived from gene expression data with gene sets^[10]^. COCOA derived conceptual foundations from this method but extends them to apply to epigenetic data and region sets. COCOA also extends beyond PCA to include other analyses including supervised analysis.

***MOGSA.*** Multi-omics gene set analysis (MOGSA) uses matrix factorization on multi-omics data from the same samples to integrate the data and reduce its dimensionality then does gene set analysis^[12]^. This method is gene-centric and not tailored to epigenetic data. As shown with MOFA in Fig. 4, multi-omics dimensionality reduction techniques could benefit from including a region-centric method such as COCOA to annotate the epigenetic component of inter-sample variation in addition to using gene set analysis

***PathwayPCA***. PathwayPCA can do pathway analysis in a variety of scenarios using supervised PCA and Adaptive Elastic-net Sparse PCA^[13]^. This method is gene-centric and is focused on pathways. As such, it has a different focus than COCOA.

***Method for DNA methylation that uses local covariation***

***CoMethDMR***. CoMethDMR is a tool to identify differentially methylated regions (DMRs)^[81]^. To boost statistical power, coMethDMR takes into account local covariation of DNA methylation within a given region. Unlike coMethDMR which uses covariation of the epigenetic signal only locally, COCOA uses covariation of the epigenetic signal on the genome-scale. While coMethDMR and COCOA may have superficial similarities, their goals are different. The output of coMethDMR is a set of differentially methylated regions while the output of COCOA is a list of region sets associated with a target variable.

**Comparison of COCOA to chromVAR**

We compared COCOA and chromVAR with two main comparisons with the breast cancer ATAC-seq data: both tools applied with the main database of region sets used for this paper and both tools applied with the curated motif database used in the chromVAR paper. For the first comparison, both methods rank ER and ER-related region sets highly although COCOA did this to a greater extent (Fig. S9A), perhaps because the use of PCA for COCOA allowed it to separate epigenetic signals more clearly. The median rank for ER region sets was 45 for PC1 of COCOA and 607 for chromVAR, with 31 ER region sets in the database. ChromVAR also did not rank hematopoietic transcription factors highly, as PC2 of COCOA did (Fig. S9A), but many of the highest scoring region sets for chromVAR were region sets for histone modifications or chromatin accessibility in immune cells. It is possible that COCOA and chromVAR are uncovering the same underlying signal but in different ways. For the second comparison, ER motifs were not ranked highly for PC1 of COCOA or for chromVAR (Fig. S9B), which may be due to differences between ER motif regions and ER ChIP-seq data, although chromVAR ranked ER motifs higher than COCOA. The median rank for ER motifs was 1309 for PC1 of COCOA and 216 for chromVAR, with 3 ER motifs in the database. Both chromVAR and PC1 of COCOA identified FOXA1 as the highest scoring ER-related motif (Fig. S9B). Both chromVAR and COCOA also identified many other FOX motifs as top results (Fig. S9C), presumably because of their similarity to FOXA1. Some of chromVAR’s highest scoring motifs were for AP1 components. AP1 colocalizes with ER and may be a tethering factor for ER^[82, 83]^. While PC1 of COCOA does not rank AP1-related motifs highly, PCs 3 and 4 do rank them highly (Fig. S9C). COCOA did not rank motifs for hematopoietic TFs highly for PC2 as it did for the region sets for hematopoietic TFs, although some hematopoietic TF motifs do have high scores for PC4 (Fig. S9C), which is more consistent with the region set results. This may once again be due to the difference between motifs and ChIP-seq region sets.

We observe that both COCOA and chromVAR achieved higher maximum scores for experimental region sets (Fig. S9A) than for motifs (Fig. S9B, S9C) although this trend could depend on the cutoff for determining motif matches (default parameters were used). For instance, the COCOA score (average absolute correlation) for PC1 for the highest ranking and median ER region sets were 0.52 and 0.40 while the scores for the highest ranking and median ER motifs were both 0.33. The chromVAR scores (standard deviation of samples’ z-scores) for the highest ranking and median ER region sets were 47.11 and 28.14 while the scores for the highest ranking and median ER motifs were 14.23 and 13.52. Because the region set database performed better for both methods, we argue that the results using the region set database are more relevant for comparing the methods. Overall, our results demonstrate that both methods can discover relevant biological insights but COCOA can separate biological signals to a greater extent than chromVAR since COCOA’s flexible framework allows the use of PCA.

**Comparison of COCOA to LOLA**

We compared COCOA to a generic region set enrichment method that does not consider covariation, LOLA^[1]^, which is a previous method associated with our lab. We performed two comparisons of COCOA and LOLA with simulated data: one in which we added a low level of noise to our samples and one in which we added a higher level of noise (Fig. S9A). Since LOLA requires a set of regions as input, we used the bumphunter R package^[84]^ to find differentially methylated regions (DMRs) between healthy and disease samples. Then, we used LOLA to test the DMRs for enrichment against our region set database. For COCOA, we performed PCA on the simulated samples then identified region sets associated with PC1 and PC2. For the comparison with a low level of noise, both methods were able to identify the region set of interest (Fig. S9B, Additional file 1: Tables S10, S11). However, with a higher level of noise, bumphunter did not identify any significant DMRs (FDR < 0.05) and we were therefore unable to run LOLA (Fig. S9C). In contrast, COCOA was still able to identify the region set of interest as relevant for PC2 (Fig. S9C, Additional file 1: Table S12). In this case, the noise apparently begins to dominate the variation among samples, and noise is therefore detected in PC1. However, the signal is still present, and is now detected by COCOA in PC2. Despite the noise, COCOA can still discover the healthy vs disease signal as relevant in PC2, while the bumphunter + LOLA approach is not able to detect significant differences. This comparison demonstrates that COCOA can better leverage the covariation of epigenetic signal to annotate epigenetic variation compared to methods that do not use covariation.


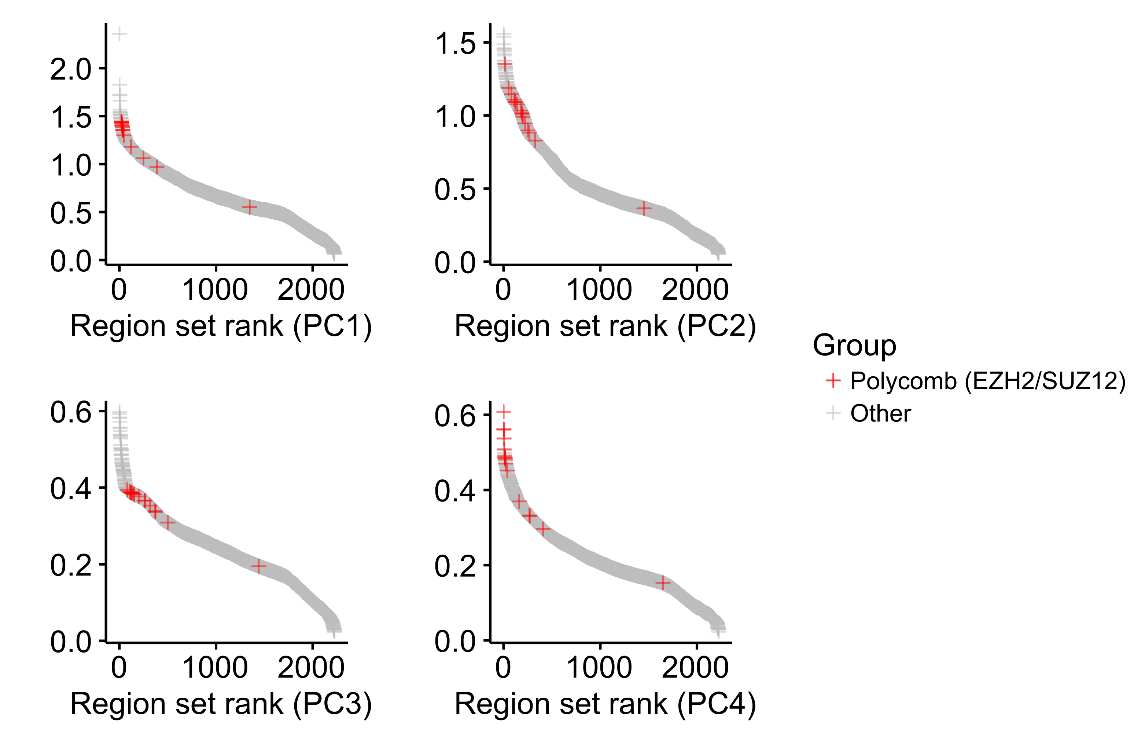


**Fig. S1. Region set scores for PCs 1-4 for the BRCA DNA methylation data.** This figure is included with only the polycomb group marked to allow clearer visualization of the polycomb region set group in comparison to Fig. 1 where several region set groups are marked.


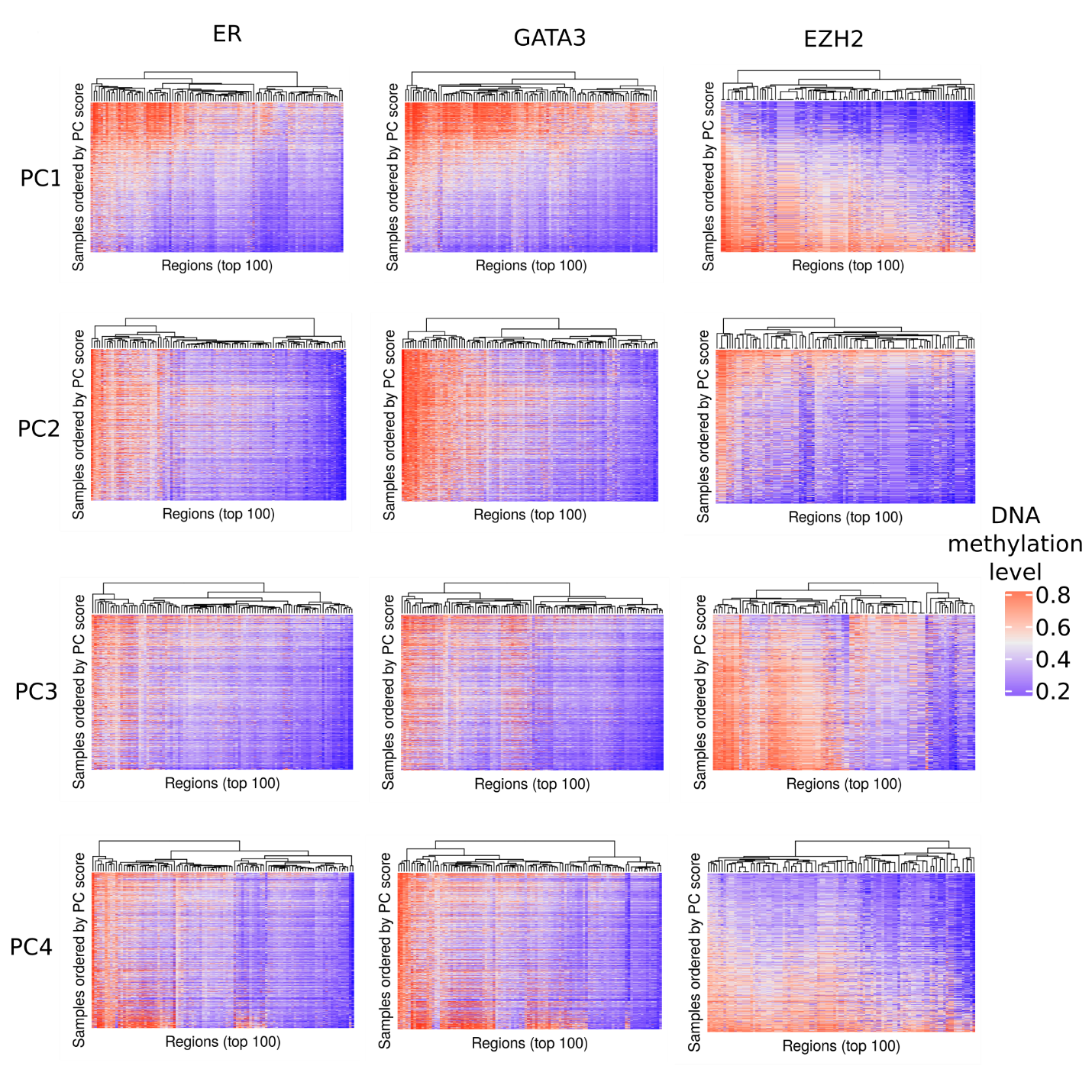


**Fig. S2. DNA methylation in some of the top scoring region sets for principal component 1.** Average DNA methylation levels are shown for the 100 regions from each region set that had the highest absolute FCS for each PC. Patients are ordered by PC scores.


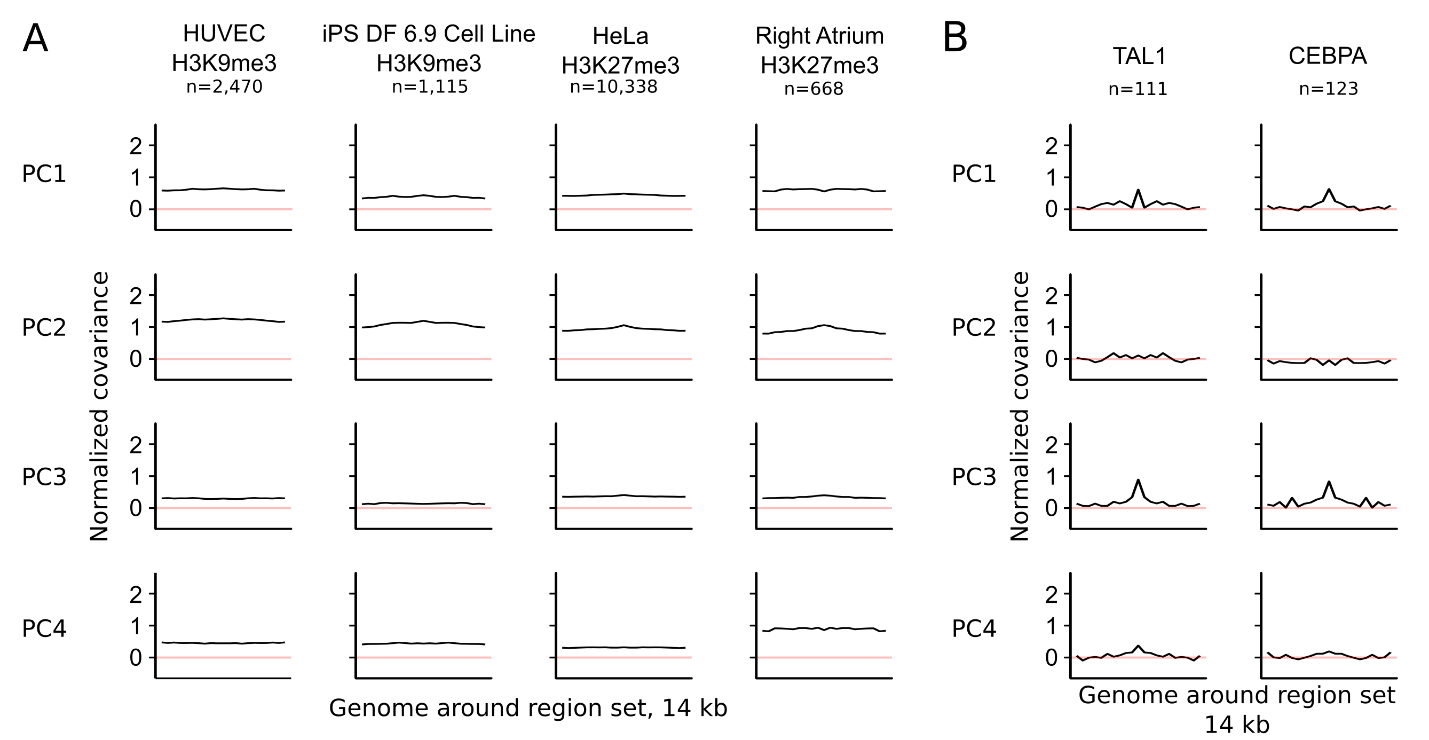


**Fig. S3. Meta-region profiles for region sets from the COCOA analysis of breast cancer DNA methylation data.** A. Profiles for the highest scoring H3K9me3 and H3K27me3 region sets from PC2. B. Profiles for the two highest scoring hematopoietic TFs in PC3. A peak in the center of the meta-region profile indicates that the DNA methylation level covaries with the PC more at the region of interest than in the surrounding genome. Profiles have been normalized to the mean and standard deviation of the covariance of all cytosines for each PC. The number of regions from each region set that were covered by the epigenetic data in the COCOA analysis (Fig. 2, panel A) is indicated by “n”.


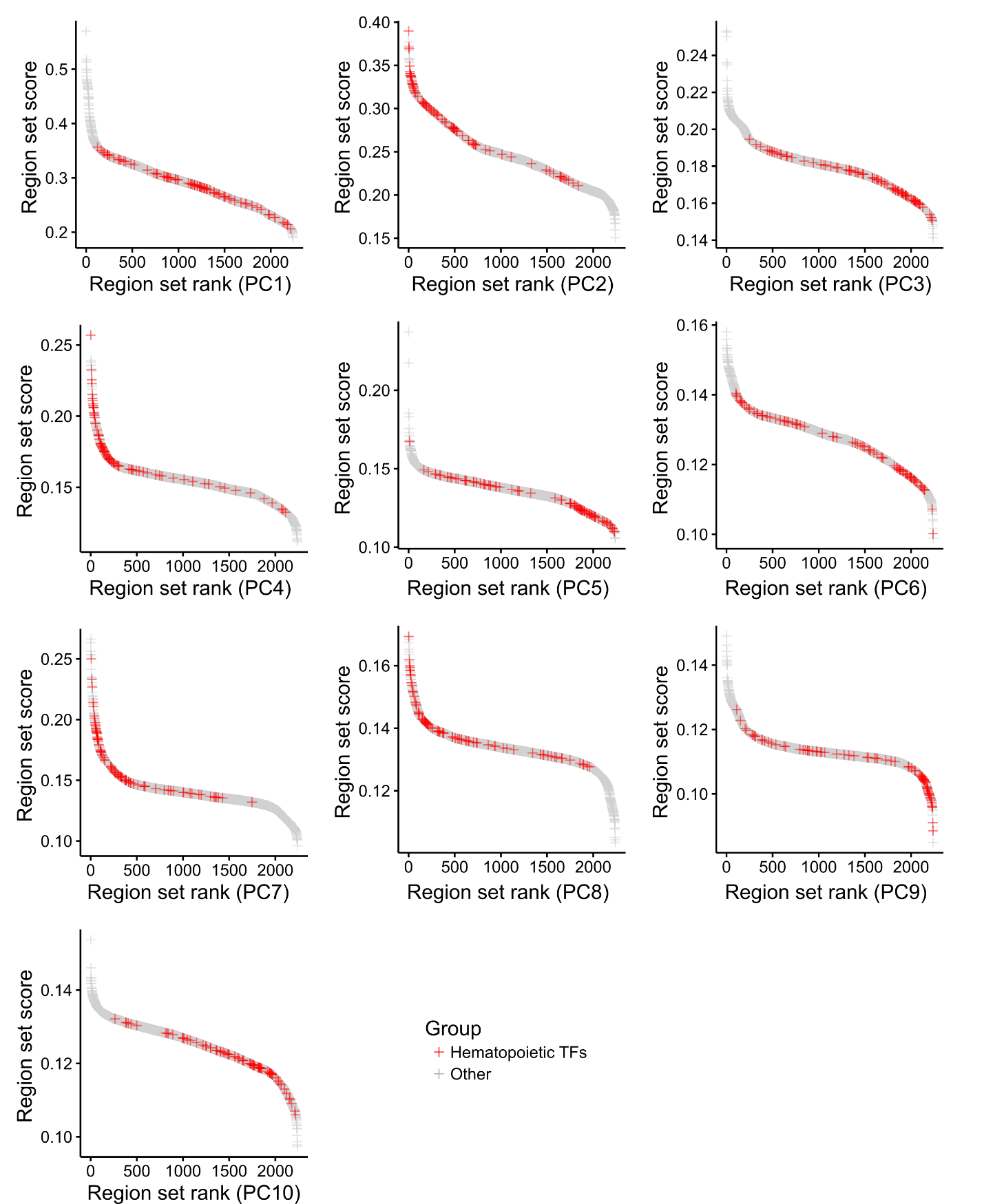


**Fig. S4. Hematopoietic transcription factor region sets have high scores for several of the top principal components.** Region set scores for each of the first 10 principal components of the BRCA ATAC-seq data.


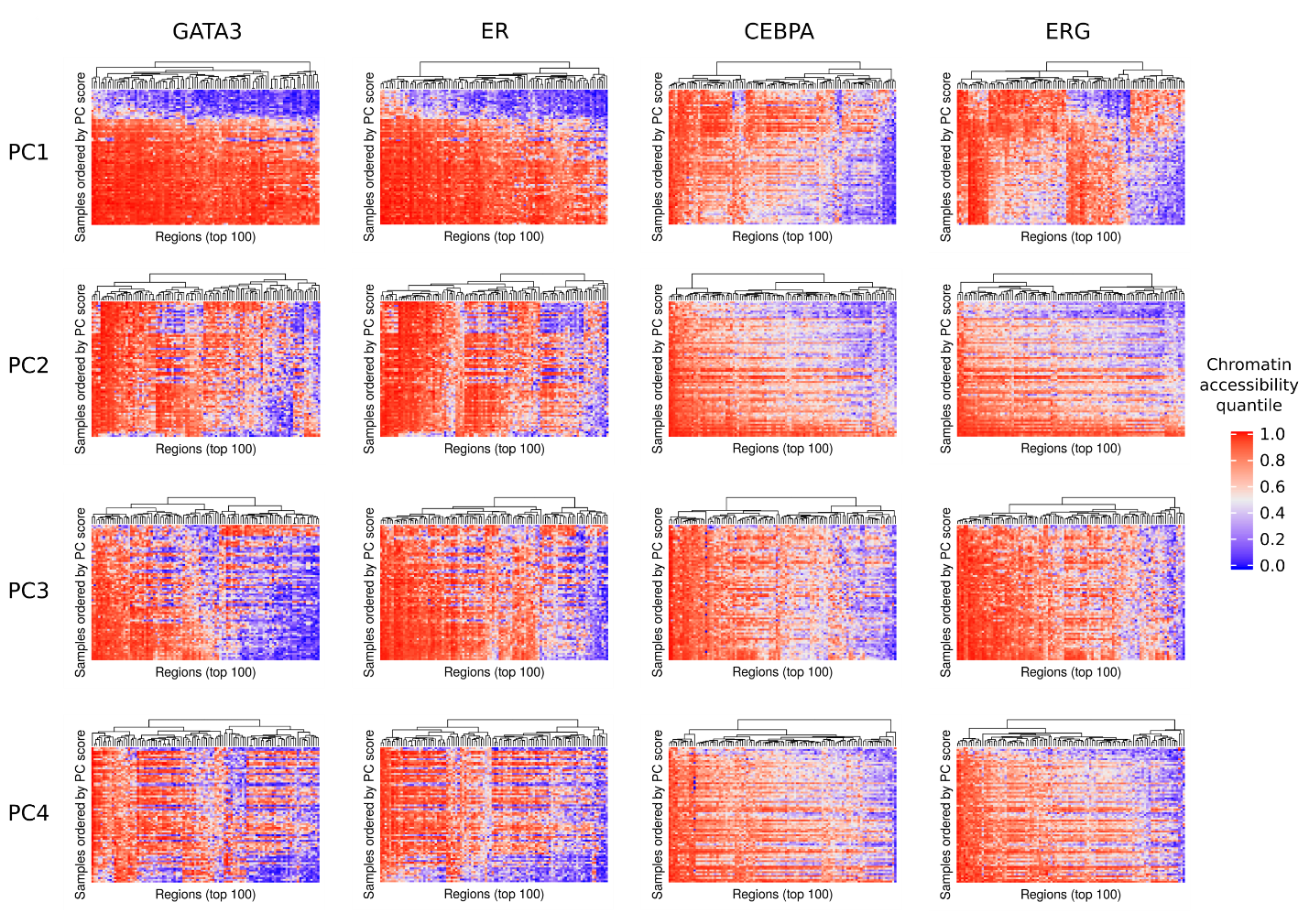


**Fig. S5. Chromatin accessibility signal in some of the top scoring region sets from COCOA analysis of breast cancer ATAC-seq data.** GATA3 and ER were the top scoring region sets for PC1 while CEBPA and ERG were the top scoring region sets for PC2. Average chromatin accessibility quantiles are shown for the 100 regions from each region set that had the highest absolute FCS for each PC. Patients are ordered by PC scores.


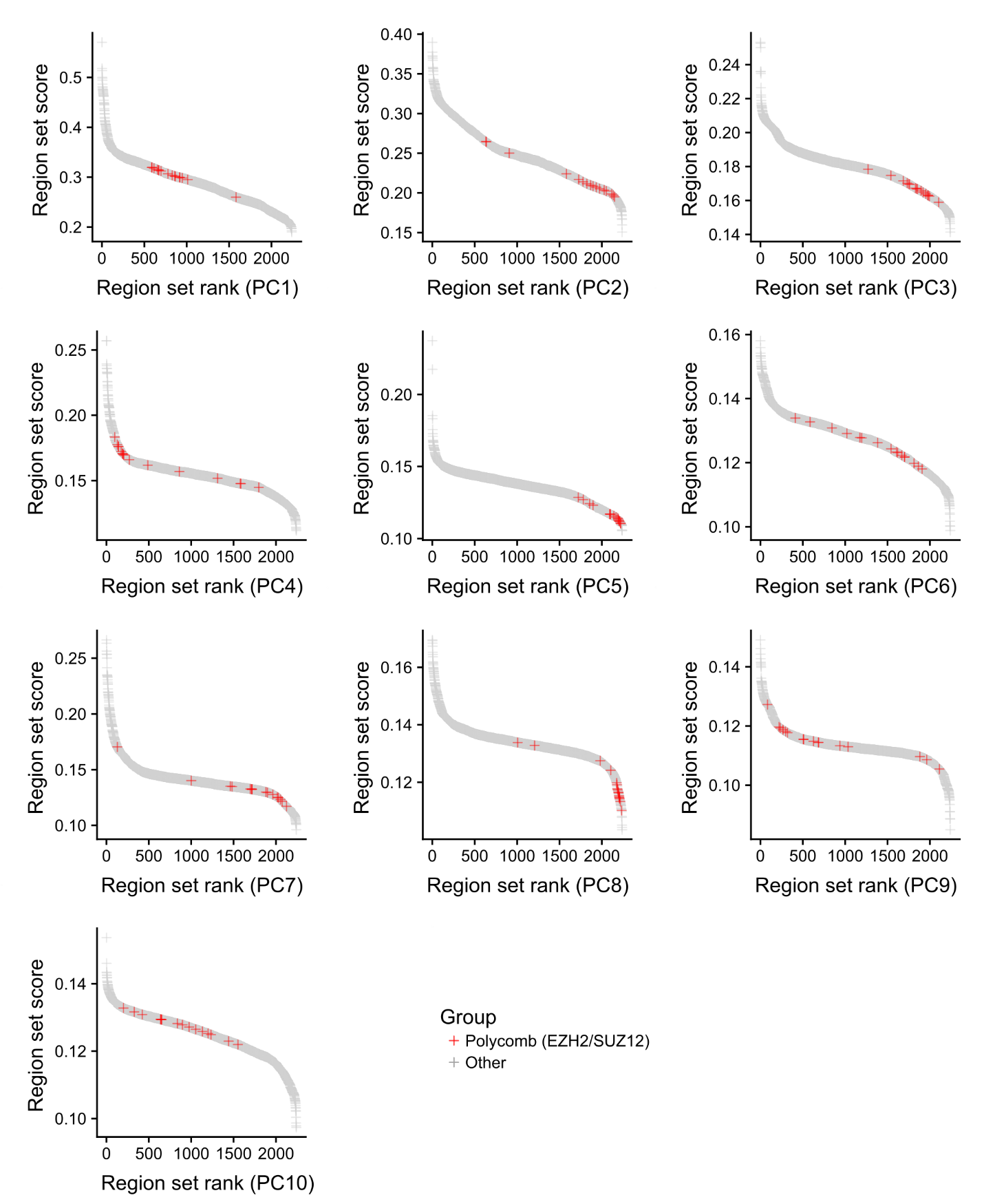


**Fig. S6. Region set scores for each of the first 10 principal components of the BRCA ATAC-seq data, with polycomb region sets (EZH2/SUZ12-binding regions) indicated.**


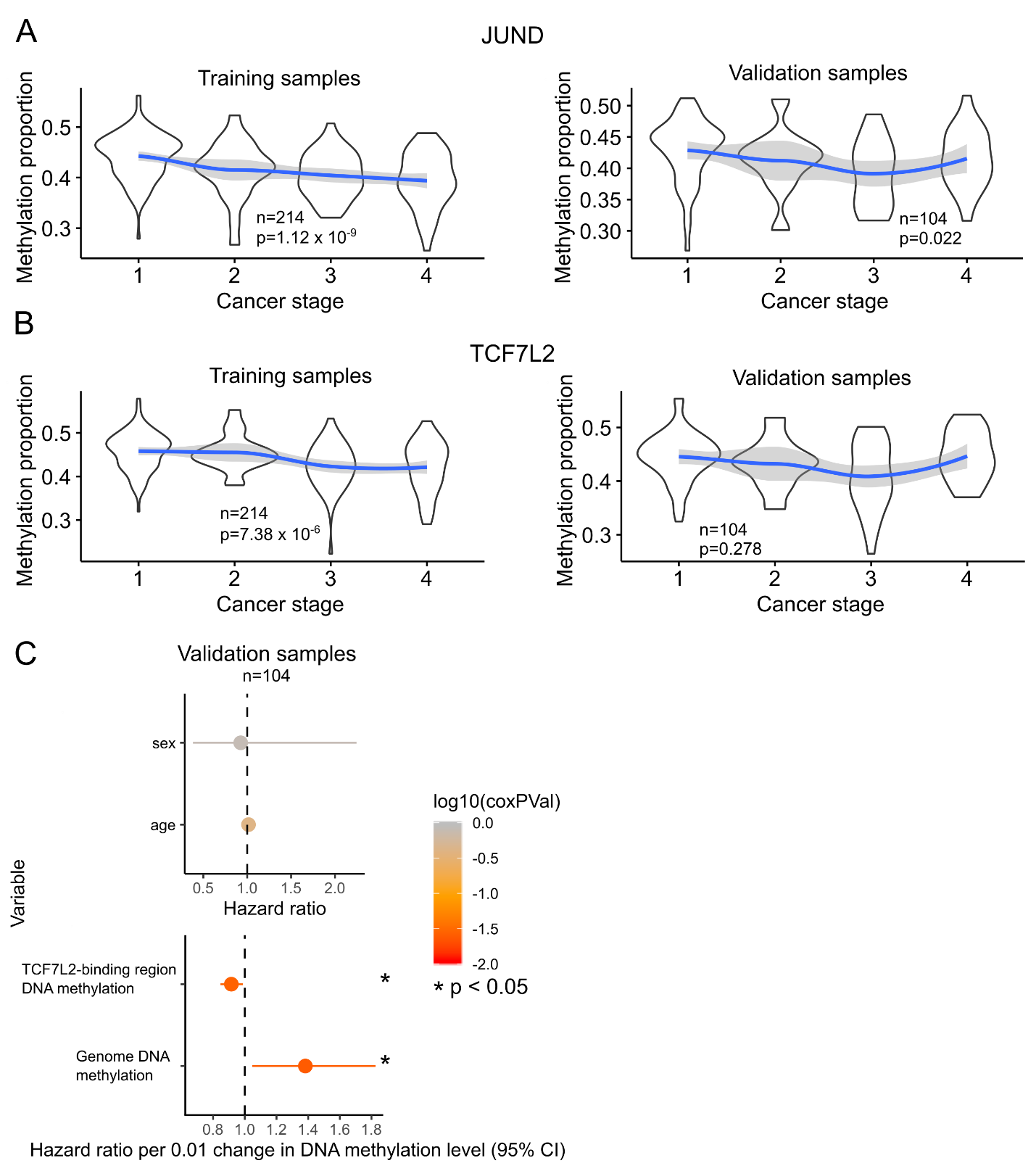


**Fig. S7. Association of average DNA methylation level in JUND and TCF7L2-binding regions with KIRC cancer stage and overall survival.** A. The Spearman correlation of cancer stage with the average DNA methylation in JUND-binding regions. The JUND region set used is the highest scoring transcription factor region set from the KIRC COCOA analysis. The JUND Cox proportional hazards model did not meet the proportional hazards assumption and is therefore not included in the figure. B. The Spearman correlation of cancer stage with the average DNA methylation in TCF7L2-binding regions. The TCF7L2 region set used is the second highest scoring transcription factor region set from the KIRC COCOA analysis. C. Hazard ratios for Cox proportional hazards model of the association between overall patient survival and average DNA methylation in TCF7L2-binding regions.


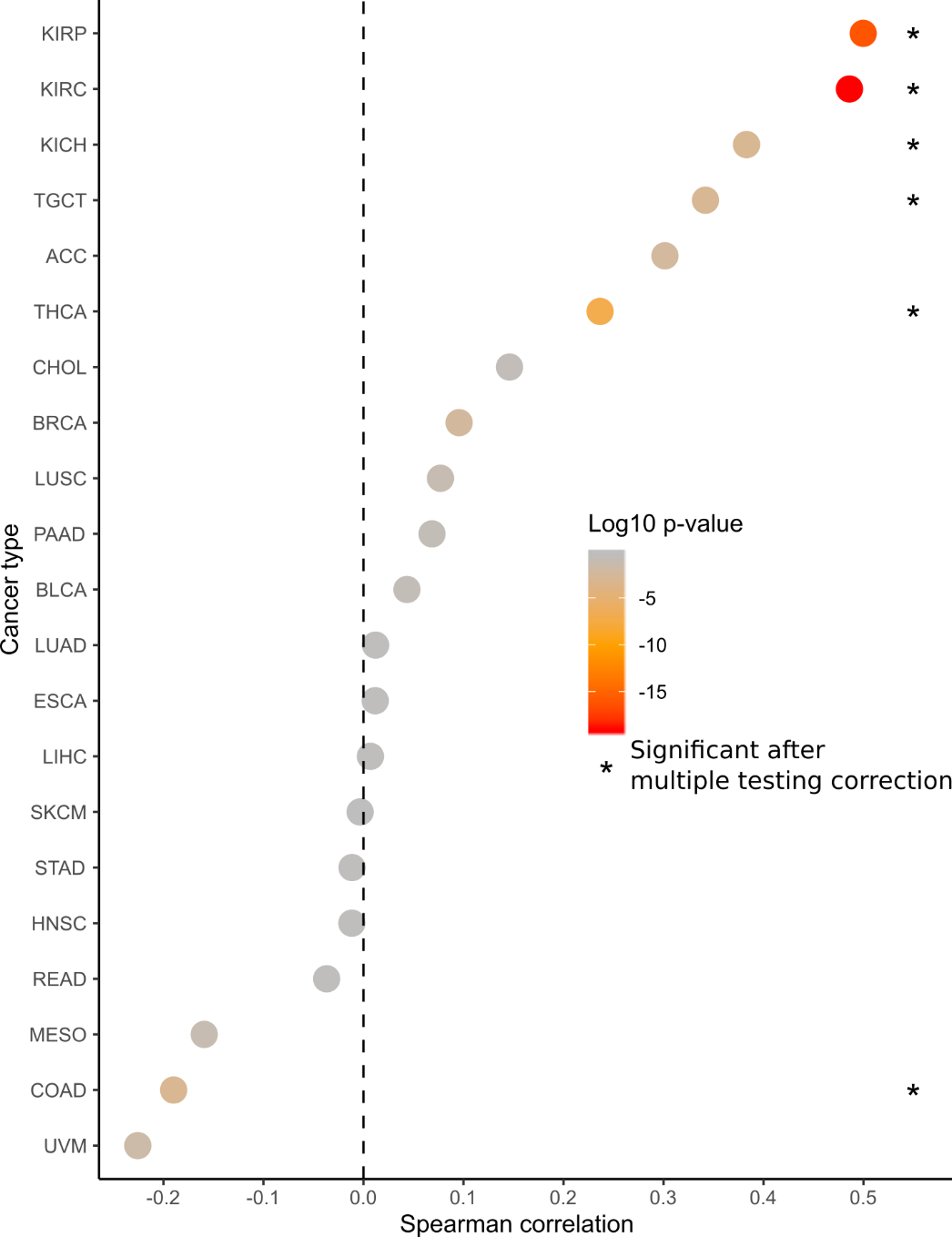


**Fig. S8. Correlation between average EZH2/SUZ12-binding region DNA methylation and cancer stage.** Color is based on the raw Spearman p-values and asterisks mark significant correlations after Holm-Bonferroni correction to account for testing 21 cancer types.

**
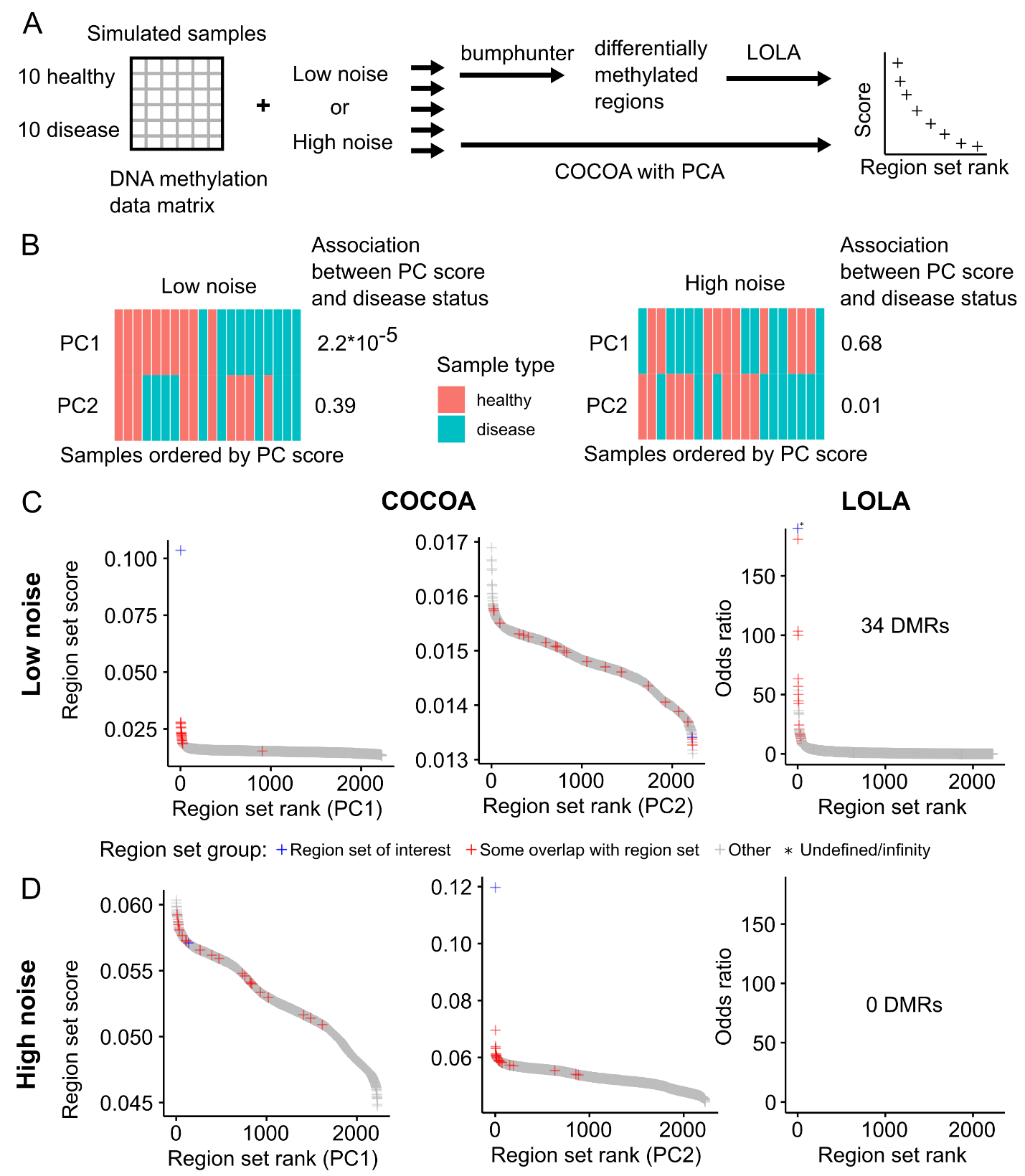
**

**Figure S9. Comparison of COCOA and LOLA.** A. The workflow for comparison of the methods. B. Association of PC scores with disease status. For low noise, PC1 is associated with disease status but for high noise, PC2 is associated with disease status (Wilcoxon rank-sum test). C. Results with a low level of noise added to samples. The COCOA score or LOLA odds ratio for each region set, ordered from highest to lowest. C. Results with a high level of noise added to samples. The COCOA score or LOLA odds ratio for each region set, ordered from highest to lowest. There are no scores for LOLA because bumphunter did not identify any significant DMRs (FDR <= 0.05).


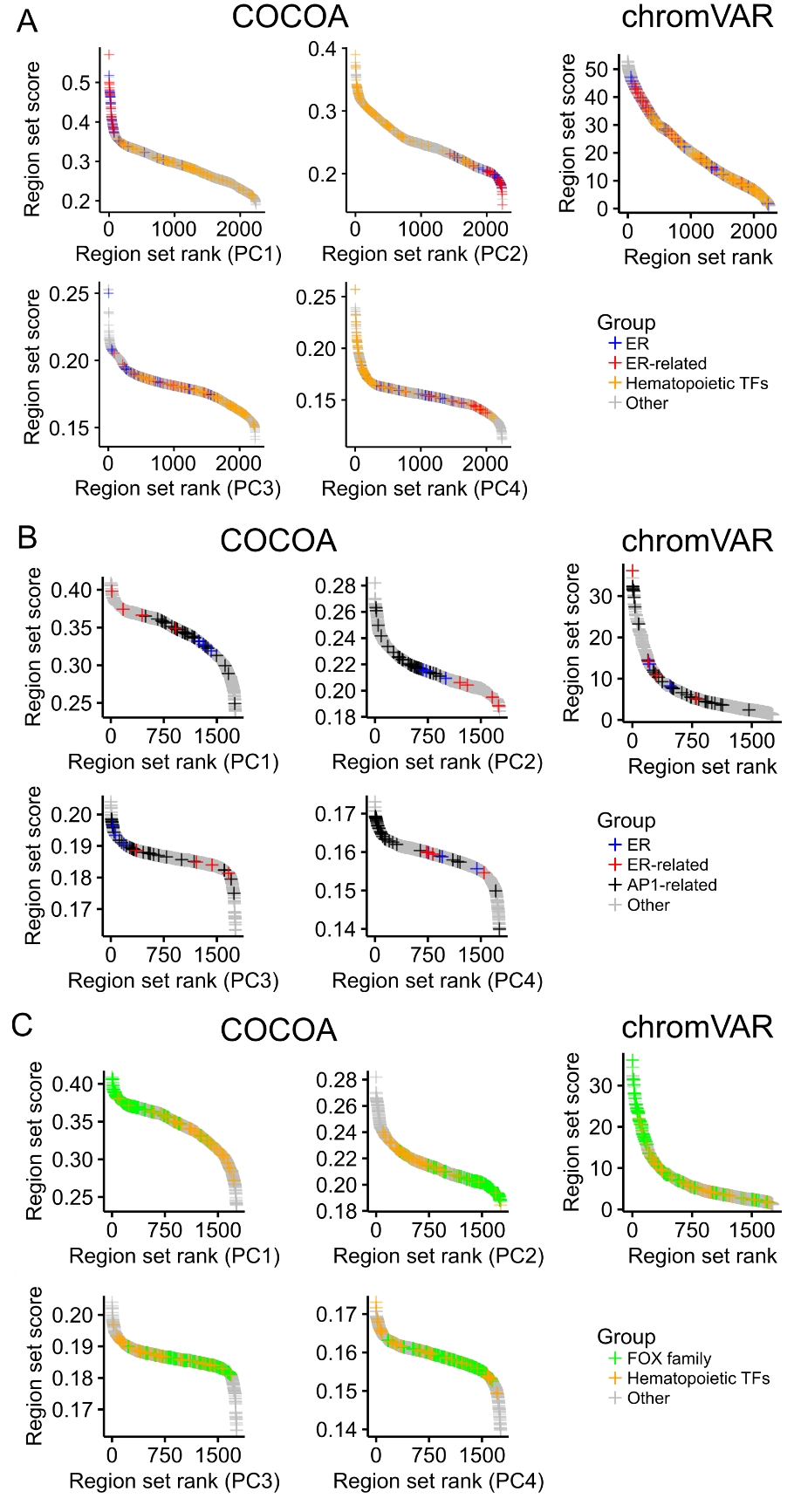


**Fig. S10. Comparison of COCOA and chromVAR on breast cancer ATAC-seq data.** A. COCOA and chromVAR scores for the region set database (see “Region set database” in methods). The chromVAR score for a region set is the standard deviation of all samples’ chromatin accessibility z-scores for that region set. The ER-related region set group includes FOXA1, GATA3, and H3R17me2. For definition of the hematopoietic TF group, see “Region set database” in methods. B. COCOA and chromVAR scores for a curated version of the cisBP motif database. The ER-related region set group includes FOXA1 and GATA3. For the definition of the AP1-related group, see *“*Comparison of COCOA and chromVAR*”* in methods. C. The same COCOA and chromVAR scores for a curated version of the cisBP motif database but indicating FOX family motifs and hematopoietic TF motifs.

**
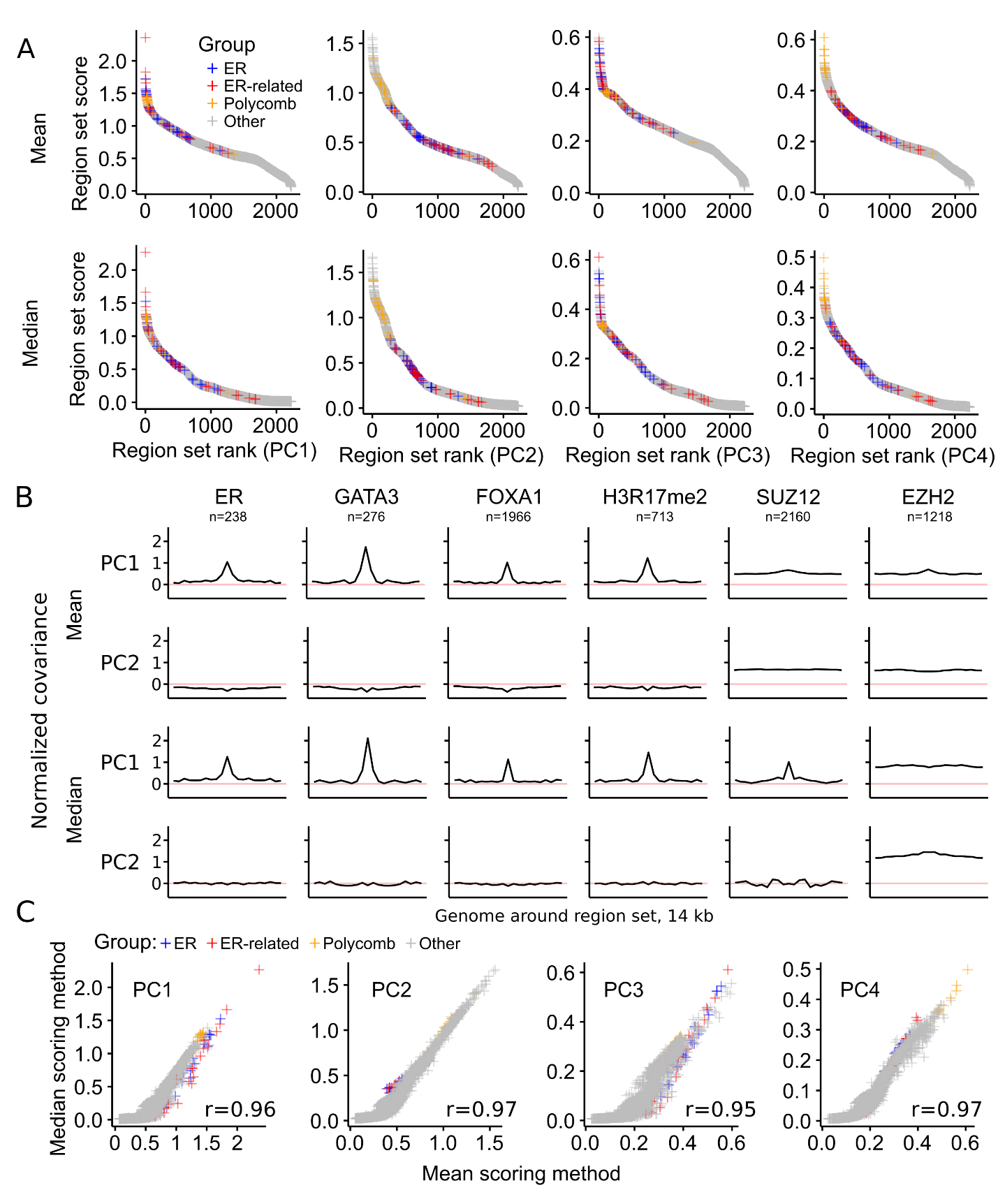
**

**Figure S11. Comparison of median and mean scoring methods.** A. The COCOA score for each region set, ordered from highest to lowest. The ER-related group includes GATA3, FOXA1, and H3R17me2. The polycomb group includes EZH2 and SUZ12. B. Meta-region profiles of several of the highest scoring region sets from the breast cancer analysis. Meta-region profiles show covariance between PC scores and the epigenetic signal in regions of the region set, centered on the regions of interest. The number of regions from each region set that were covered by the epigenetic data in the COCOA analysis (panel A) is indicated by “n”. The line at zero marks the mean or median respectively of the FCS for each PC. C. The relationship between region set scores for each scoring method. The Spearman correlation is shown.

**
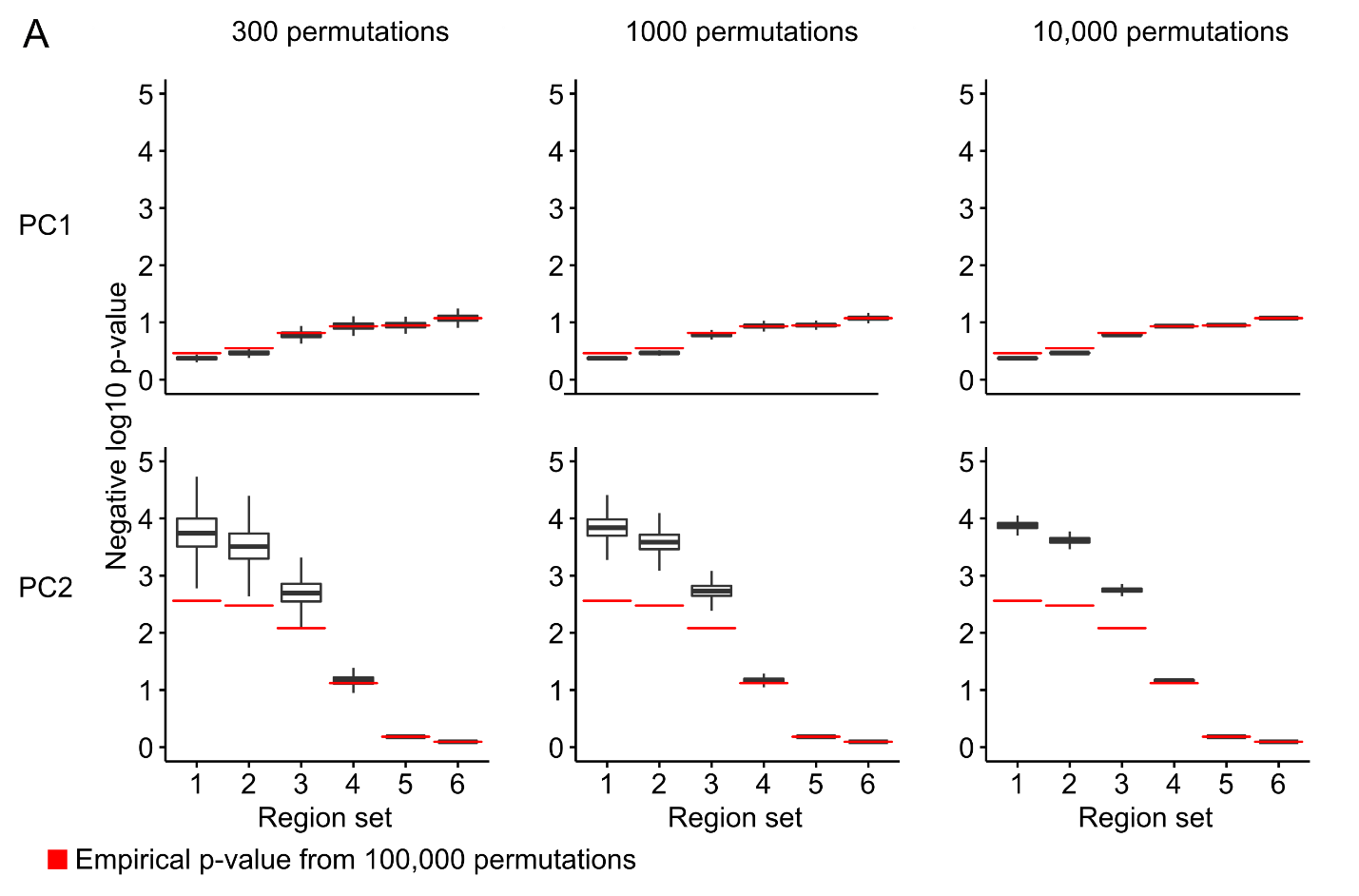
**

**Figure S12. Comparison of empirical p-values to gamma distribution p-value approximation.** COCOA was run on PC1 and PC2 of PCA of simulated data with six region sets. The empirical p-values from 100,000 permutations are shown. P-values were also calculated with a gamma distribution approximation after sampling either 300, 1000, or 10,000 permutations from the 100,000 that were calculated. 500,000 such samples were taken to get a distribution of gamma p-values for each region set (outliers not shown).

**References**

70. Ma S, Ogino S, Parsana P, Nishihara R, Qian Z, Shen J, et al. Continuity of transcriptomes among colorectal cancer subtypes based on meta-analysis. Genome Biology. 2018;19(1).

71. Chikina MD, Troyanskaya OG. An effective statistical evaluation of ChIPseq dataset similarity. Bioinformatics. 2012;28(5):607–13.

72. Breeze CE, Reynolds AP, van Dongen J, Dunham I, Lazar J, Neph S, et al. eFORGE v2.0: updated analysis of cell typespecific

signal in epigenomic data. Bioinformatics. 2019;35(22):4767–9.

73. Yu G, Wang LG, He QY. ChIPseeker: an R/Bioconductor package for ChIP peak annotation, comparison and visualization. Bioinformatics. 2015;31(14):2382–3.

74. Wang Z, Civelek M, Miller CL, Sheffield NC, Guertin MJ, Zang C. BART: a transcription factor prediction tool with query gene sets or epigenomic profiles. Bioinformatics. 2018;34(16):2867–9.

75. Hansen KD, Irizarry RA, WU Z. Removing technical variability in RNA-seq data using conditional quantile normalization. Biostatistics. 2012;13(2):204–16.

76. Hinz S, Magheli A, Weikert S, Schulze W, Krause H, Schrader M, et al. Deregulation of EZH2 expression in human spermatogenic disorders and testicular germ cell tumors. World J Urol. 2009;28(5):631–5.

77. Singh R, Fazal Z, Corbet AK, Bikorimana E, Rodriguez JC, Khan EM, et al. Epigenetic remodeling through downregulation of polycomb repressive complex 2 mediates chemotherapy resistance in testicular germ cell tumors. Cancers. 2019;11(6):796.

78. Suva ML, Riggi N, Janiszewska M, Radovanovic I, Provero P, Stehle JC, et al. EZH2 is essential for glioblastoma cancer stem cell maintenance. Cancer Res. 2009;69(24):9211–8.

79. Cheng T, Xu Y. Effects of enhancer of zeste homolog 2 (EZH2) expression on brain glioma cell proliferation and tumorigenesis. Med Sc Monit. 2018;24:7249–55.

80. Farlik M, Halbritter F, Muller F, Choudry FA, Ebert P, Klughammer J, et al. DNA methylation dynamics of human hematopoietic stem cell differentiation. Cell Stem Cell. 2016;19(6):808–22.

81. Gomez L, Odom GJ, Young JI, Martin ER, Liu L, Chen X, et al. coMethDMR: accurate identification of co-methylated and differentially methylated regions in epigenome-wide association studies with continuous phenotypes. Nucleic Acids Res. 2019;47(17):e98.

82. He H, Sinha I, Fan R, Haldosen LA, Yan F, Zhao C, et al. c-Jun/AP-1 overexpression reprograms ER signaling related to tamoxifen response in ER-positive breast cancer. Oncogene. 2018;37(19):2586–600.

83. Miranda TB, Voss TC, Sung MH, Baek S, John S, Hawkins M, et al. Reprogramming the chromatin landscape: interplay of the estrogen and glucocorticoid receptors at the genomic level. Cancer Res. 2013;73(16):5130–9.

84. Jaffe AE, Murakami P, Lee H, Leek JT, Fallin MD, Feinberg AP, et al. Bump hunting to identify differentially methylated regions in epigenetic epidemiology studies. Int J Epidemiol. 2012;41(1):200–9.
