# Supplementary material for: COCOA: coordinate covariation analysis of epigenetic heterogeneity
Source: Genome Biol. 2020 Sep 7;21:240. doi: 10.1186/s13059-020-02139-4 (PMC7487606; doi:10.1186/s13059-020-02139-4)
Supplement: Supplementary file 10 — Additional file 10. Review history. [file 13059_2020_2139_MOESM10_ESM.docx]

Review History

**First round of review**

**Reviewer 1**

**Comments to author:**

Lawson et al. presented a new methodology called Coordinate Covariation Analysis (COCOA) which uses covariation of epigenetic signals across individuals to identify and annotate epigenetic heterogeneity. The manuscript is well written, and the tool will be useful for users to interpret the many DNA methylation studies that have been (or will be) conducted. However, some technical details need to be better assessed and discussed before the paper is ready for prime time.

Major comments

1. In the Methods section (page 18, line 388-391), it is stated “Within each region from the region set, we average the FCS of any overlapping features to get a single average value for each region. We then average the region scores to get the final score for that combination of region set and FCS vector”.

Here the average was used twice in the algorithm to derive a score for each sample, so the algorithm might be influenced by outlier values. A better alternative might be the median. It would be informative for users if some assessment of impact of outlier samples and comparison of results using medians vs. mean or some other methods for summarizing sample specific score can be presented. At the very least, these should be implemented as alternative options in the software.

2. How does the COCOA algorithm compare to enrichment type of methods? For example, LOLA presented by the authors previously. In principle, LOLA can also annotate association of DNA methylation data with phenotype respect to region sets, using Fisher’s exact test.

3. In Methods section (page 19, line 435-438), it’s stated ”To reduce the computational burden, we calculated 300 permutations and applied a permutation approximation technique. We fit a gamma distribution to each null distribution using the method of moments in the fitdistrplus R package and then calculated a p-value for each region set using its gamma distribution”.

How does the gamma distribution approximation work in this type of data? It would be informative for users if the authors can provide some empirical results on how good this approximation is (e.g. quantile-quantile plot), in what situation the approximation works well and in what situation users should avoid the approximation.

Minor comments

1. More details on the use of COCOA should be included in discussion. For example, in Supplementary information (page 17, line 378-380), it is stated “Here, we have used a subset of the LOLA database[1], which includes several thousand region sets that have been manually collected from several large-scale experiments and databases, including the ENCODE[39, 40] and Roadmap Epigenomics projects [41, 42].”

Why were the ENCODE and Roadmap region sets chosen instead of other region sets? Are these the ones the users should always use? Also some consideration on the cell types should be discussed, as DNA methylation data is cell type specific.

2. Multiple transcription factors often work together synergistically, along with co-factors to influence target gene expressions. How might that influence the results of COCOA algorithm?

3. What sample sizes are need for the algorithm to work well? What types of outcomes can be analyzed – binary, continuous, or survival outcome? If binary outcomes, how many samples in each group are needed?

**Reviewer 2**

**Comments to author:**

Lawson et al present COCOA, a novel annotation enrichment analysis method for high-dimensional omics data that aims to depart from associating binarized signals with differences observed in discrete sample groups towards quantitative correlation-based enrichment scores. Briefly, based on a data-base of annotated genomic regions COCOA identifies region sets showing a significant association with a target variable of interest. For an “unsupervised” scenario in which such variable is not given, authors use alternative surrogate variables, e.g. principal components. Authors demonstrate the utility of their methods on several single- and multi-omics data-sets, including a DNA methylation and ATAC-seq data of breast cancer, as well as mutli-omics profiles of CLL samples.

While the results of COCOA application to various data sets appear biologically meaningful, the major problem of the manuscript is that the benefits of COCOA have not been clearly demonstrated with respect to standard and generic approaches for both, supervised and unsupervised scenarios.

1)	For the supervised scenario, how does the performance of COCOA compare to the canonical approach, i.e. first determining features significantly associated with the target variable of interest, be it binary, categorical or quantitative, and performing region annotation enrichment analysis, say with the author’s own earlier tool LOLA to keep the database of sets comparable. Will these two approaches yield different enriched region-sets?

2)	Similarly, in an unsupervised variant of their method, authors use principal components as one possible surrogate for a target phenotype. The utility of the method should be compared to a more generic and straightforward approach, e.g. summarizing PCA loadings for each region set. Will the ranking be different in this case, as compared to the one generated by COCOA? While the authors compare their results to gene-centric approach in the multi-omics scenario, this is not an equivalent scenario.

Performing these two benchmarks is crucial to unambiguously demonstrate the added value of COCOA over the commonly used approaches. A feature-based comparison to alternative tools which the authors did carry out is in fact superficial and hence insufficient. Although there are hardly any real data sets that would provide ground truth for such a comparison, it should be relatively uncomplicated to design several synthetic examples with simulated perturbations of defined region sets.

**Authors Response**

**Point-by-point responses to the reviewers’ comments:**

Reviewer 1

Lawson et al. presented a new methodology called Coordinate Covariation Analysis (COCOA) which uses covariation of epigenetic signals across individuals to identify and annotate epigenetic heterogeneity. The manuscript is well written, and the tool will be useful for users to interpret the many DNA methylation studies that have been (or will be) conducted. However, some technical details need to be better assessed and discussed before the paper is ready for prime time.

*Thank you for your positive comments! We are glad you found COCOA useful and appreciate your critiques below, which have improved the manuscript and the method.*

Major comments

1. In the Methods section (page 18, line 388-391), it is stated “Within each region from the region set, we average the FCS of any overlapping features to get a single average value for each region. We then average the region scores to get the final score for that combination of region set and FCS vector”.

Here the average was used twice in the algorithm to derive a score for each sample, so the algorithm might be influenced by outlier values. A better alternative might be the median. It would be informative for users if some assessment of impact of outlier samples and comparison of results using medians vs. mean or some other methods for summarizing sample specific score can be presented. At the very least, these should be implemented as alternative options in the software.

*Thank you for the comment, this is a good idea. To address the concern about outliers, we have now added the option to score based on the median instead of the mean as you requested. In addition, we also tested the median scoring method on the breast cancer DNA methylation data and compared to our previous results with the mean, which we have added to the paper as a supplemental figure (Fig. S11). Briefly, we found consistent overall results and region set scores displayed high correlation between the two methods in this case. However, we agree that this result will depend on the data, so adding the median-based approach will improve COCOA results on other datasets. We added a detailed discussion of this topic in the Supplementary Information, in which we caution users to be wary of outlier samples and advise to try the median-based approach in such cases.*

2. How does the COCOA algorithm compare to enrichment type of methods? For example, LOLA presented by the authors previously. In principle, LOLA can also annotate association of DNA methylation data with phenotype respect to region sets, using Fisher’s exact test.

*COCOA and LOLA are similar in the sense that they both rely on databases of region sets; however, there are many differences. LOLA takes as input a region set – nothing more than a BED file with chromosome, start, and stop columns. It attempts to identify region sets from the database that are most similar. As such, it is a more generic tool. COCOA is a more sophisticated method, which operates on actual signal data across a population of samples. So, for COCOA the input is not a region set, but a matrix of annotated samples with genomic signal data of some kind. As such, COCOA as a population-based approach, while LOLA requires a user to summarize the data into a single region set for analysis. Furthermore, COCOA runs computations on the actual signal, using all available data; it does not rely on a pre-selection of a subset of regions.*

*One way to use LOLA to do a similar type of analysis to COCOA was also pointed out below by reviewer 2. In this analysis, a user first summarizes the population signal data into a region set, for example, by calling differential methylation peaks using an external method. Then, this peak set can be put into LOLA to identify which region sets are similar. As raised by reviewer 2, this type of approach has a similar overall goal to COCOA and is one of many possible applications of the more generic LOLA. However, it does not take into account the covariation of the epigenetic signal, and also relies on calling discrete differential regions, which causes information loss. Please see comments below, where we describe how we have comprehensively addressed this in the revision, including a simulation study comparing the two approaches directly.*

3. In Methods section (page 19, line 435-438), it’s stated ”To reduce the computational burden, we calculated 300 permutations and applied a permutation approximation technique. We fit a gamma distribution to each null distribution using the method of moments in the fitdistrplus R package and then calculated a p-value for each region set using its gamma distribution”.

How does the gamma distribution approximation work in this type of data? It would be informative for users if the authors can provide some empirical results on how good this approximation is (e.g. quantile-quantile plot), in what situation the approximation works well and in what situation users should avoid the approximation.

*We appreciate this comment, as it has prompted us to more thoroughly explore the p-value approximation. In response, we have restructured some of the COCOA software to make the permutations significantly faster. This will facilitate the calculation of more accurate p-values going forward. However, even with these algorithmic improvements, it is still time consuming to run a permutation test so we have retained the gamma approximation. To directly address the question of applicability, we did a simulation study to assess the appropriateness of the gamma approximation. We ran a simulation with a high number of permutations and then subsampled and applied the approximation to see how close the approximation is to the true p-value. Our conclusion is that the gamma approximation is accurate for high p-values, but that it tends to predict lower p-values than the empirical p-values as the p-value decreases (Figure S12, and new supplementary discussion). This has the effect of making p-values look smaller than they should be for very small p-values. Overall, we are satisfied that the approximation, while not perfect, is still useful, and we have now added a section discussing the gamma distribution approximation to the Supplementary Information. In this section, we caution users to treat the result as an approximation, particularly when the p-values become very low.*

Minor comments

1. More details on the use of COCOA should be included in discussion. For example, in Supplementary information (page 17, line 378-380), it is stated “Here, we have used a subset of the LOLA database[1], which includes several thousand region sets that have been manually collected from several large-scale experiments and databases, including the ENCODE[39, 40] and Roadmap Epigenomics projects [41, 42].”

Why were the ENCODE and Roadmap region sets chosen instead of other region sets? Are these the ones the users should always use? Also some consideration on the cell types should be discussed, as DNA methylation data is cell type specific.

*Thank for you the suggestion – we have added a section to the Supplementary Information about considerations when choosing a region set database. As you suggest here, the COCOA method is dependent on the choice of database. Briefly, the region sets we used from ENCODE, Roadmap Epigenomics, and other sources provide a reasonably broad sampling of transcription factors and histone modification regions for a variety of cell lines and tissue types. We do not consider this a suggestion for the gold standard region sets to use, simply a (somewhat arbitrary) decision that turned out to work for our project. More research definitely needs to be done in optimizing the choice of databases, which is likely to be different for different biological questions.*

*Most important, we have designed the software to be flexible enough to allow the user to construct and use any database of interest. COCOA will perform better if a user takes time to create a database with relevant region sets for a particular biological question. The curation of region set databases is an active research area and a moving target due to the continual release of region set data from new studies. The database we used in this paper is available as a useful starting point that can be expanded in the future. A comprehensive region set database would be very valuable and we and others are continuing to work on approaches for handling the ever-growing corpus of public region set data.*

2. Multiple transcription factors often work together synergistically, along with co-factors to influence target gene expressions. How might that influence the results of COCOA algorithm?

*If regions where epigenetic signal covaries across samples are regulated by multiple TFs, then all the TFs will likely be found to be significant, depending on their degree of overlap with the varying regions. A benefit of this approach is that COCOA can identify multiple proteins involved in the same regulatory program. However, COCOA does not assert that an identified protein is the only regulator of a set of regions or that the protein is causally linked to the epigenetic variation.*

3. What sample sizes are need for the algorithm to work well? What types of outcomes can be analyzed – binary, continuous, or survival outcome? If binary outcomes, how many samples in each group are needed?

*This is an excellent question, but a difficult one to answer concretely because there are many influencing factors at play. The number of samples needed depends both on the number of regions with epigenetic covariation and the average effect size of the epigenetic variation. In our hands, we had as few as 73 samples in our ATAC-seq analyses, which yielded clearly biologically meaningful results. Furthermore, in our synthetic (binarized) dataset we identified the region set of interest with only 20 samples.*

*Both binary and continuous outcomes can be analyzed. COCOA only requires that each genomic locus be given a score representing how much the epigenetic signal at that locus is associated with the outcome. When analyzing binary outcomes, the user may miss potentially relevant epigenetic variation between samples with the same label. COCOA can analyze survival data as binary or continuous outcomes but COCOA has not been designed to deal with censored survival data. It would be possible in the future to add the option to COCOA to give feature contribution scores (FCS) using a metric that does account for censored survival data such as a univariate Cox proportional hazards model but we anticipate that approach would not scale well when computing permutations. However, users have the ability to compute FCS themselves and give them as input to COCOA so users could do a survival analysis with the current software, if they desired.*

Reviewer #2

Lawson et al present COCOA, a novel annotation enrichment analysis method for high-dimensional omics data that aims to depart from associating binarized signals with differences observed in discrete sample groups towards quantitative correlation-based enrichment scores. Briefly, based on a data-base of annotated genomic regions COCOA identifies region sets showing a significant association with a target variable of interest. For an “unsupervised” scenario in which such variable is not given, authors use alternative surrogate variables, e.g. principal components. Authors demonstrate the utility of their methods on several single- and multi-omics data-sets, including a DNA methylation and ATAC-seq data of breast cancer, as well as multi-omics profiles of CLL samples.

While the results of COCOA application to various data sets appear biologically meaningful, the major problem of the manuscript is that the benefits of COCOA have not been clearly demonstrated with respect to standard and generic approaches for both, supervised and unsupervised scenarios.

*Thank you for the positive comments about COCOA and for your thoughtful review. We agree with your assessment and have addressed this with a new simulation study that more clearly demonstrates the value of COCOA, which we describe below.*

1) For the supervised scenario, how does the performance of COCOA compare to the canonical approach, i.e. first determining features significantly associated with the target variable of interest, be it binary, categorical or quantitative, and performing region annotation enrichment analysis, say with the author’s own earlier tool LOLA to keep the database of sets comparable. Will these two approaches yield different enriched region-sets?

*Indeed, this is a great idea to compare, and similar to a question raised by reviewer 1. We have now done a detailed comparison using a simulated dataset. Briefly, the conclusion is that if the signal is strong enough, COCOA and LOLA will yield similar, but not identical results. However, if the signal is weaker, eventually, the differential analysis will begin to be unable to call differences accurately, causing the signal to be diluted and weakening the results of the enrichment-based approach. As signal deteriorates, COCOA becomes more and more useful as it is able to skip the discrete step of calling differential regions. This analysis strengthens the argument of COCOA’s utility, which leverages the increased information to provide more robust results for its particular use case.*

*To more fully explain the strengths of COCOA in this analysis setting, we have now added a new section and supplemental figure (Figure S9) to the manuscript that describes the results of a simulated analysis run using each approach. We demonstrate the strength of using COCOA, which leverages covariation of epigenetic signal across samples which can give a stronger signal, which is lost in the two-step approach of differential analysis + LOLA. In our comparison, we see that both methods work in the presence of low noise but COCOA is superior when signal is weaker (Fig. S9). We have also added a comparison of LOLA and COCOA to the Supplementary Information.*

2) Similarly, in an unsupervised variant of their method, authors use principal components as one possible surrogate for a target phenotype. The utility of the method should be compared to a more generic and straightforward approach, e.g. summarizing PCA loadings for each region set. Will the ranking be different in this case, as compared to the one generated by COCOA? While the authors compare their results to gene-centric approach in the multi-omics scenario, this is not an equivalent scenario.

*Thank you for the suggestion, this comment has caused us to carefully think about the COCOA approach and consider the source of its strengths. The analysis you suggest is in fact exactly what early versions of the COCOA software did. Our more recent developments have extended this approach, but essentially, what you describe is the basis of what COCOA does when using principle components as the target variables. To demonstrate, we ran COCOA on the breast cancer DNA methylation data we previously analyzed, now using this basic mode to summarize PCA loadings for each region set. The rankings are perfectly correlated with the ones presented in the paper, which uses only a slightly modified version of this approach (described below). In our experience, this general approach is not widespread; while most unsupervised analysis of DNA methylation and other genomic signals starts with a some kind of dimensionality reduction such as PCA, the task of annotating the reduced dimensions is quite variable, and done in different ways or omitted, and using region set aggregation is not typical. Our original effort on COCOA was intended to simply this very task, and is still possible to run as a base mode in COCOA. As we developed COCOA, we have added some useful features that extend this approach: First, it is generic to the dimensionality reduction method. So, in fact, users can take any approach to generate the target variables that passed to COCOA, not just PCA. Second, COCOA’s correlation-based approach allows us to do permutation tests in a more reasonable amount of time, while only running the dimensionality reduction approach a single time. To compute permutations otherwise would require running PCA for each permutation. Third, it allows us to extend COCOA to datasets where the dimensionality reduction was only calculated on a subset of the data. For example, in the MOFA analysis presented in the paper, only a small subset of CpGs are used to calculate the dimensionality reduction. COCOA allows us to use all the data even with these reduced analyses by correlating the original data to the PC/latent factor. Finally, COCOA allows us to use different similarity metrics. Using PCA loadings directly (which is also allowed by COCOA) is roughly equivalent to using Pearson correlation if each variable was normalized to variance of one before the PCA or equivalent to covariance if variance normalization was not performed; but in the paper we suggested that it is sometimes more appropriate to use other metrics such as Spearman correlation. In practice, COCOA is flexible and any similarity function can be used, providing flexibility for different data types and analysis approaches. In short, our paper emphasizes the value of using covariation in epigenetic analysis, clearly bringing into focus an underutilized principle of epigenetics. While a few other methods also recognize this principle (see Table 1), we offer a generalized framework through our software and a first-of-its-kind tool for DNA methylation, along with many methodological improvements, so that the broader community can easily take advantage of this principle.*

Performing these two benchmarks is crucial to unambiguously demonstrate the added value of COCOA over the commonly used approaches. A feature-based comparison to alternative tools which the authors did carry out is in fact superficial and hence insufficient. Although there are hardly any real data sets that would provide ground truth for such a comparison, it should be relatively uncomplicated to design several synthetic examples with simulated perturbations of defined region sets.

*We greatly appreciate these two comments and hope that our revised manuscript addresses your concern and convinces you that COCOA is a very flexible framework with meaningful advances that will contribute to analysis of epigenetic heterogeneity. We agree that our earlier comparisons are a bit superficial, but this is out of necessity, because the methods are not directly comparable. Nevertheless, on the surface, they may appear similar to the casual reader, so we think that a general guideline about the similarities and differences among methods is useful for many readers.*

**Second round of review**

**Reviewer 1**

The authors adequately addressed my questions and concerns.

**Reviewer 2**

Thanks, both of my comments were sufficiently addressed.

Taking an opportunity to express this suggestion for the future development, both author's tools COCOA and LOLA would profit from regular maintenance and expansion of their region set databases. Possible interesting regions that could be included are refined catalogues of repetitive sequences, hotspots of oncogenic mutations (e.g. from TCGA and PCAWG projects) etc
